# Effectiveness of the 10-Valent Pneumococcal Nontypeable *Haemophilus influenzae* Protein D–Conjugated Vaccine (PHiD-CV) Against Carriage and Acute Otitis Media—A Double-Blind Randomized Clinical Trial in Finland

**DOI:** 10.1093/jpids/piw010

**Published:** 2016-04-28

**Authors:** Timo Vesikari, Aino Forsten, Ilkka Seppä, Tarja Kaijalainen, Taneli Puumalainen, Anu Soininen, Magali Traskine, Patricia Lommel, Sonia Schoonbroodt, Marjan Hezareh, Marta Moreira, Dorota Borys, Lode Schuerman

**Affiliations:** 1 Vaccine Research Centre, University of Tampere Medical School; 2 National Institute for Health and Welfare, Oulu; 3 GSK, Espoo, Finland; 4 GSK Vaccines, Wavre, Belgium

**Keywords:** acute otitis media, nasopharyngeal carriage, PHiD-CV, pneumococcal conjugate vaccine, *Streptococcus pneumoniae*

## Abstract

After administering the 10-valent pneumococcal polysaccharide nontypeable *Haemophilus influenzae* protein D–conjugated vaccine (PHiD-CV) to children aged 2–18 months, we observed a reduction in vaccine-type nasopharyngeal carriage, resulting in a reduction of overall pneumococcal nasopharyngeal carriage, which may be important for indirect vaccine effects. We noted a trend toward reduction of acute otitis media.

**Background:**

This trial (ClinicalTrials.gov identifier NCT00839254), nested within a cluster-randomized double-blind invasive pneumococcal disease effectiveness study in Finland (ClinicalTrials.gov identifier NCT00861380), assessed the effectiveness of the 10-valent pneumococcal polysaccharide nontypeable *Haemophilus influenzae* protein D–conjugated vaccine (PHiD-CV or PCV10) against bacterial nasopharyngeal carriage and acute otitis media (AOM).

**Methods:**

Infants (aged 6 weeks to 6 months) received the PHiD-CV or a control vaccine (hepatitis B) (schedule 3+1 or 2+1). Nasopharyngeal swabs were collected at 4 time points post-vaccination from all of the infants and at pre-vaccination from a subset. Parent-reported physician-diagnosed AOM was assessed from first vaccination until last contact (mean follow-up, 18 months). Vaccine effectiveness (VE) was derived as (1 – relative risk)*100, accounting for cluster design in AOM analysis. Significant VE was assessed descriptively (positive lower limit of the non-adjusted 95% confidence interval [CI]).

**Results:**

The vaccinated cohort included 5093 infants for carriage assessment and 4117 infants for AOM assessment. Both schedules decreased vaccine-serotype carriage, with a trend toward a lesser effect from the 2+1 schedule ( VE across timpoints 19%–56% [3+1] and 1%–38% [2+1]). Trends toward reduced pneumococcal carriage (predominantly vaccine serotypes 6B, 14, 19F, and 23F), decreased carriage of vaccine-related serotype 19A, and small increases at later time points (ages 14–15 months) in non–vaccine-serotype carriage were observed. No effects on nontypeable *Haemophilus influenzae, Staphylococcus aureus*, or *Moraxella catarrhalis* carriage were observed. There were non-significant trends toward a reduction in the number of infants reporting AOM episodes (VE 3+1: 6.1% [95% CI, −2.7% to 14.1%] and 2+1: 7.4% [−2.8% to 16.6%]) and all AOM episodes (VE 3+1: 2.8% [−9.5% to 13.9%] and 2+1: 10.2% [−4.1% to 22.9%]). PHiD-CV was immunogenic and had an acceptable safety profile.

**Conclusions:**

We observed reduced vaccine-type pneumococcal carriage, a limited increase in non–vaccine-type carriage, and a trend toward AOM reduction.

## INTRODUCTION

*Streptococcus pneumoniae* is a leading cause of respiratory tract infections and bacterial invasive disease [1]. Bacterial nasopharyngeal carriage precedes infection, and various studies have pointed toward a causal link between carriage and disease [2]. Children younger than 5 years are a population vulnerable to pneumococcal disease, and they form a reservoir for other age groups. Reduced carriage of *S pneumoniae* decreases exposure of unvaccinated individuals, which results in substantial indirect effects [2, 3].

*S pneumoniae* is one of the main bacterial pathogens in acute otitis media (AOM). In Finland, estimations of AOM incidence vary from 370 to 630 per 1000 child-years [4] to >1100 per 1000 child-years [5]. Clinical trials have shown efficacy of pneumococcal conjugate vaccines (PCVs) against vaccine-type AOM, but they have generally shown little or no efficacy against all-cause AOM (vaccine efficacy range, 1%–7%) [5–9]; except for one study showing efficacy of 34% for an 11-valent pneumococcal protein D–conjugated vaccine in children [10].

The 10-valent pneumococcal polysaccharide non-typeable *Haemophilus influenzae* (NTHi) protein D–conjugated vaccine (PHiD-CV, or PCV10) [11–13] was licensed in the European Union in March 2009 (*Synflorix™*, GSK Vaccines). In addition to the recommended 3+1 schedule, PHiD-CV has increasingly been administered in a 2+1 schedule when given as part of routine infant immunization programs; however, efficacy/effectiveness data for the 2+1 schedule are limited. FinIP, a large cluster-randomized study in Finland, was the first clinical trial in Europe to document the effectiveness of PHiD-CV against invasive pneumococcal disease (IPD) and the impact on outpatient antimicrobial purchases when administered as a 2+1 or 3+1 schedule [14, 15]. Here, we present results from a nested study that evaluated the effectiveness of PHiD-CV, given on different schedules, against nasopharyngeal carriage as an indication of the potential to induce herd effects. This is the largest carriage assessment study with PHiD-CV to date. We also evaluated the effectiveness of PHiD-CV against AOM (in parallel to another clinical trial in Latin America [16]) and PHiD-CV immunogenicity and safety.

## METHODOLOGY

### Study Design and Participants

This phase III double-blind trial (ClinicalTrials.gov identifier NCT00839254), conducted between February 2009 and December 2011 in 15 study clinics in Finland coordinated by the Tampere University Vaccine Research Centre, was nested within the larger cluster-randomized FinIP study (ClinicalTrials.gov identifier NCT00861380), which assessed the effectiveness of PHiD-CV against IPD [14]. In addition to the FinIP effectiveness objectives, this study evaluated PHiD-CV immunogenicity, safety, and effectiveness against carriage and AOM.

Children aged 6 weeks to 18 months who had not received a pneumococcal vaccine, a hepatitis A or B vaccine, or any investigational or nonregistered product and who had no contraindications to immunization were eligible for enrollment. Enrollment ended when PHiD-CV was introduced into the Finnish National Vaccination Program (NVP) (September 2010); before then, there had been limited PCV use.

The study was conducted in accordance with Good Clinical Practice principles and the Declaration of Helsinki. The protocol was approved by an independent ethics committee. For each participant, written informed consent was obtained from each patient's parent(s) or legal guardian(s).

The study is registered at ClinicalTrials.gov (NCT00839254) and available at http://www.gsk-clinicalstudyregister.com/study/112595?study_ids=112595#ps).

### Study Vaccines and Procedures

Participants received the PHiD-CV or a control vaccine (hepatitis B [*Engerix-B™*] for children <12 months of age or hepatitis A [*Havrix™ 720 Junior*] for children ≥12 months of age [both provided by GSK Vaccines]). PHiD-CV contains 10 serotype-specific pneumococcal polysaccharides conjugated to *H influenzae* protein D, tetanus toxoid, or diphtheria toxoid [14].

**Figure 1.**
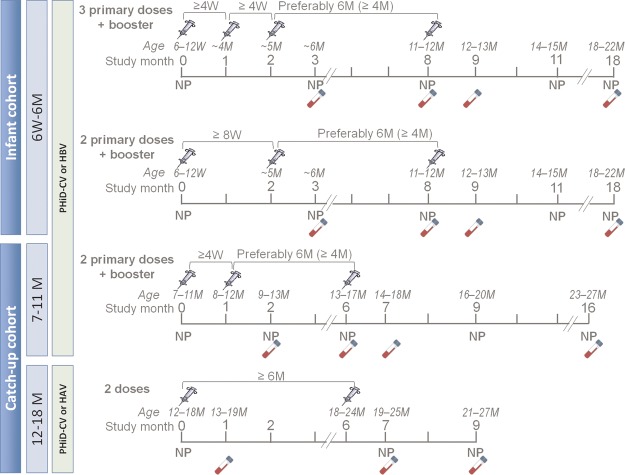
Study design. Syringes indicate vaccination; vials indicate blood sample acquisition. Abbreviations: HAV, hepatitis A vaccine; HBV, hepatitis B vaccine; M, months; NP, nasopharyngeal swab; PHiD-CV, 10-valent pneumococcal polysaccharide nontypeable Haemophilus influenzae protein D–conjugated vaccine; W, weeks.

Participants received study vaccines according to an age-appropriate schedule: the 2+1 or 3+1 schedule for children aged 6 weeks to 6 months at enrollment (infant cohorts); the 2+1 schedule for children aged 7–11 months at enrollment (7- to 11-month catch-up cohort); or 2 doses for children aged 12–18 months at enrollment (12- to 18-month catch-up cohort) (Figure 1). Routine pediatric vaccines, such as the diphtheria, tetanus, acellular pertussis, and inactivated polio virus/*H influenzae* type B (DTaP–IPV/Hib) and human rotavirus vaccines, were coadministered at 3 and 5 months of age; the DTaP–IPV/Hib vaccine was also coadministered at 11–12 months.

### Randomization

Clusters were randomized (2:2:1:1: PHiD-CV 3+1, PHiD-CV 2+1, control 3+1, control 2+1) using a blocking scheme, stratified according to cluster size (below/above average), urbanity (urban/rural), and Tampere University Vaccine Research Centre trial enrollment. For nested study participants, individual randomization codes were used, aligned with cluster randomization based on place of residence.

### Outcomes

Study outcomes included PHiD-CV effect on *S pneumoniae* colonization (including all pneumococcal serotypes, vaccine serotypes, non-vaccine/non–vaccine-related serotypes, and vaccine-related serotypes, particularly 6A and 19A) and other bacteria (NTHi, *Moraxella catarrhalis*, and *Staphylococcus aureus*).

Study outcomes also included all-cause AOM and all-cause AOM with antimicrobial prescription. We assessed effectiveness in reducing the number of children reporting ≥1 AOM episode, and in reducing the occurrence of all AOM episodes. We also evaluated PHiD-CV safety and reactogenicity for all participants and immunogenicity for a subset of them (see Supplementary Methods).

### Carriage Assessment

Study personnel collected nasopharyngeal samples from all participants using a pediatric rayon-tipped swab at the post–primary vaccination and post-booster visits (Figure 1). Pre-vaccination swabs were collected from the infant immunogenicity subset and from all children in the catch-up cohorts. All samples were transferred to STGG (skim milk, tryptone, glucose, and glycerol) transport medium [17] and stored below –65°C before transport to the laboratory at the National Institute for Health and Welfare in Oulu, Finland. A detailed description of culture, identification, and serotyping is provided in Supplementary Methods.

### AOM Assessment

Parents were asked by automated text message every 2 weeks if their child had had a physician-confirmed AOM diagnosis. If there was no reply, a reminder message was sent after 24 hours; after 48 hours, the parents were contacted by a study nurse by telephone. If no contact could be made, AOM status was checked at the next study visit.

For cases reported by the parents as physician-confirmed AOM, regardless of documentation in the medical records or other source documents, parents were asked to report AOM and antimicrobial prescriptions in an AOM questionnaire. Finland's national guidelines recommend antibiotics, when AOM diagnosis is certain [18], which are only available upon prescription.

### Statistical Analysis

The encompassing FinIP study was powered to show significant differences (α = .05) in the rate of vaccine-type IPD between the PHiD-CV 3+1 and control groups in the infant cohort. The nested study reported here was not designed to draw any formal statistical conclusions, but it allowed descriptive assessments of the AOM, carriage, safety, and immunogenicity objectives without predefined success criteria. For carriage, assuming 1200 evaluable children per group and a 12.2% incidence rate of vaccine-type carriage in the control group, the study had 80% power to detect a vaccine effectiveness (VE) of 37%. For AOM, assuming 4500 evaluable children in the infant cohort (randomized 1:1:1) and an AOM incidence rate of 0.55 in the control group, the study had 80% power to detect a VE of 19.6%.

Informative conclusions on statistical significance of the effectiveness were based on the positive lower limit of its non-adjusted 95% confidence interval (CI) and should be interpreted with caution linked to the descriptive character of the end points.

Carriage and safety analyses were performed for the total vaccinated cohort (TVC), which comprised all children who received ≥1 vaccine dose according to treatment actually received. The percentage of participants with a positive nasopharyngeal sample and the 95% CI were calculated, as were VE, estimated as (1 – relative risk)*100, with the 95% CIs, derived using a conditional exact method. Across-visits results include the pre-vaccination visit.

We also evaluated cumulative acquisition, defined as the occurrence of bacterial pathogens or serotypes not detected at any of the previous time points; VE were calculated with 95% CIs (Supplementary Table 4). For the infant cohort, as pre-vaccination swabs were collected only from the immunogenicity subset, cumulative acquisition from pre- to 1 month post-primary vaccination was analyzed separately (Supplementary Table 4). For the full infant cohort, the first cumulative acquisition data from infants at the age of 11–12 months are presented, and 1 month post-primary vaccination (infant age 6 months) was the reference time point.

AOM analyses were performed for the TVC for AOM effectiveness (excluding misrandomized children who did not receive the treatment assigned to their cluster). A new AOM episode was defined if it occurred ≥30 days after onset of the previous episode. We report results for AOM with level 1 diagnostic certainty: parent-reported physician-diagnosed AOM in the infant cohort. The number of participants in the catch-up cohorts was too low to obtain meaningful results. The analyzed follow-up time for the infant TVC started on the day of first vaccination and ended at the infants' last visit (planned at 18–22 months of age). A negative binomial model taking into account the cluster effect and stratification factors was used to derive VE against AOM as (1 – relative risk)*100 with 95% CIs, as detailed previously [14].

Blood samples were planned to be collected for the approximately 1500 first enrolled participants (immunogenicity subset). Immunogenicity analyses were performed on the according-to-protocol immunogenicity cohort, which comprised all evaluable subset participants (who met all eligibility criteria, complied with protocol-defined procedures/intervals, and met no elimination criteria) with results from ≥1 assay available.

Statistical analyses were performed using Statistical Analysis Systems (SAS Institute, Inc., Cary, North Carolina) version 9.22 or SAS Drug Development (SDD) and the StatXact-8.0 procedure (Cytel Software Corp, Cambridge, Massachusetts) on SAS.

## RESULTS

### Study Participants

**Figure 2.**
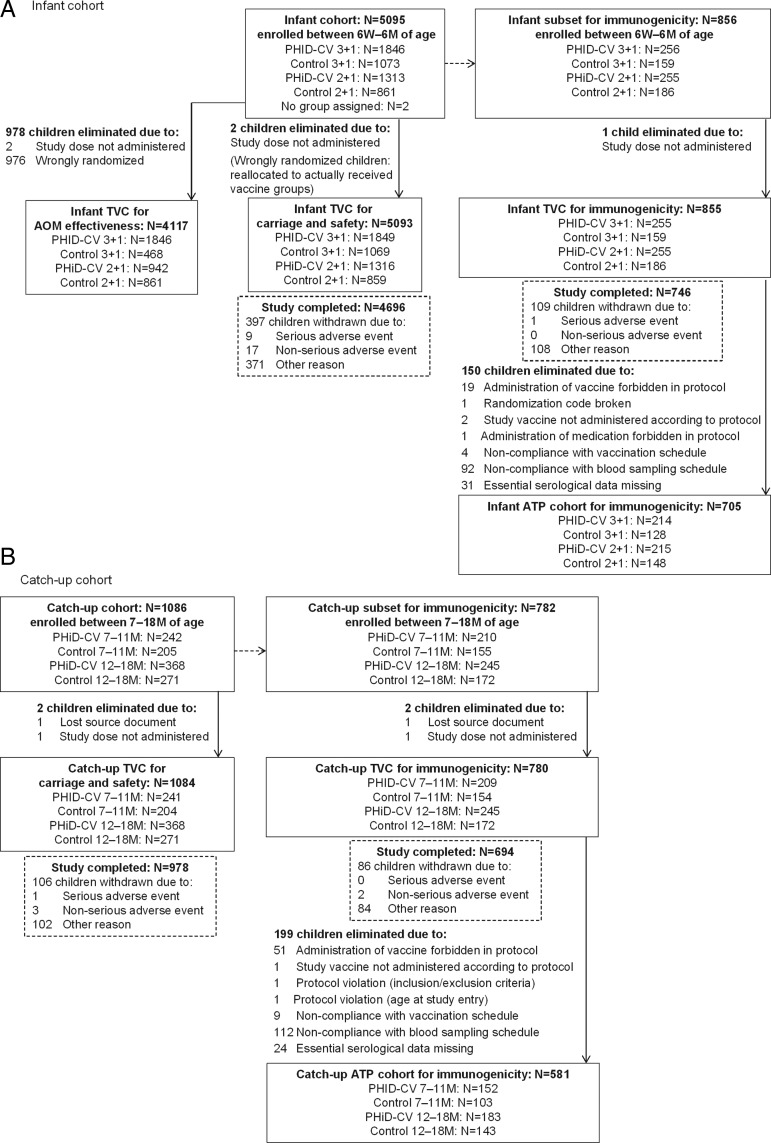
Participant flow chart. Because of an error in treatment number allocation, 3 children had 2 subject numbers allocated each; thus, the actual number of enrolled children was 6178 instead of 6181, corresponding to 5092 children instead of 5093 in the infant total vaccinated cohort for carriage/safety and 1082 instead of 1084 in the catch-up total vaccinated cohort for carriage/safety. Data for these children were recorded only once for the subject number corresponding to the time of participation. Abbreviations: AOM, acute otitis media; ATP, according-to-protocol; M, months; N, number of children in the specified group; PHiD-CV, 10-valent pneumococcal polysaccharide nontypeable Haemophilus influenzae protein D–conjugated vaccine; TVC, total vaccinated cohort; W, weeks.

A total of 6178 infants and toddlers were enrolled in 50 clusters (Figure 2). Demographic characteristics were comparable between the PHiD-CV and control groups (Supplementary Table 1). The mean follow-up time was 18 months. The immunogenicity subset comprised 1635 children (855 infants and 780 toddlers; Figure 2).

Because of a randomization error, 976 infants did not receive the treatment assigned to their cluster. These misrandomized infants were reallocated to the groups corresponding to the vaccination they actually received for the TVC for carriage/safety and immunogenicity according-to-protocol cohort (analyses per individual randomization) but were excluded from the TVC for AOM effectiveness (cluster-randomized analysis), which substantially affected the 2+1 PHiD-CV group for AOM assessment (371 misrandomized children) [14].

### Effect of PHiD-CV Vaccination on Nasopharyngeal Carriage of *S pneumoniae*

#### Infant Groups

**Figure 3.**
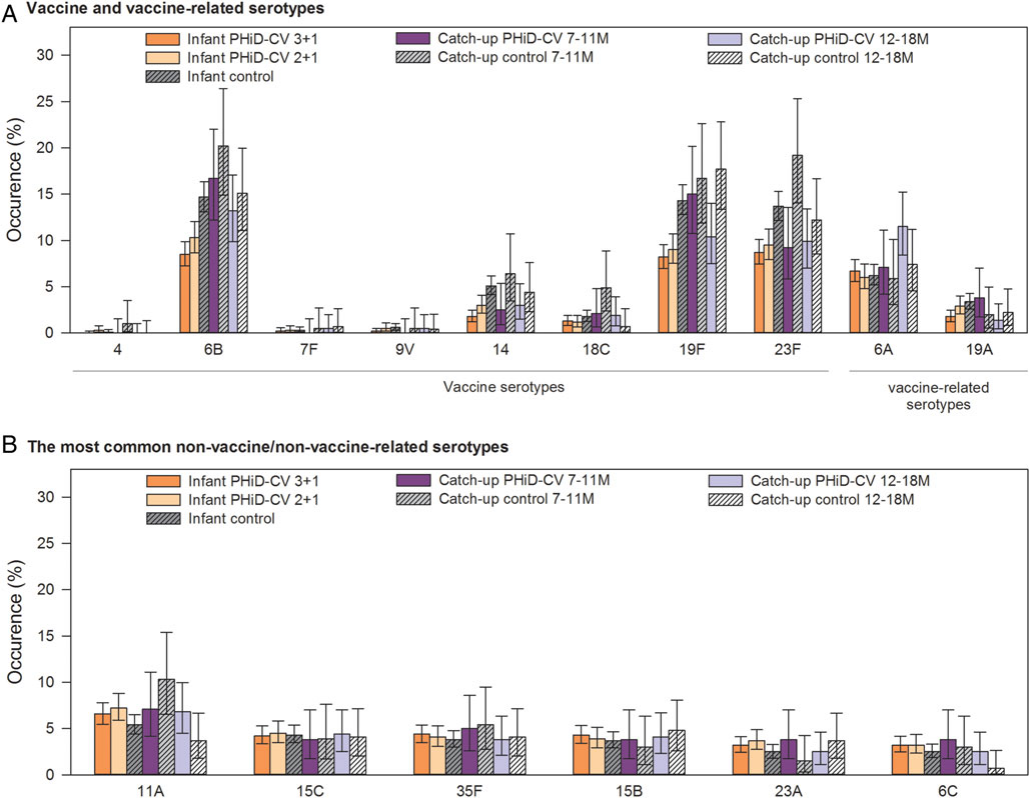
Percentage of children with nasopharyngeal colonization across all visits (total vaccinated cohort for carriage). The occurrence of *S pneumoniae* serotypes in nasopharyngeal swabs across all visits (including baseline) is shown. No carriage for vaccine serotype 1 and 5 was observed. Abbreviations: M, months; PHiD-CV, 10-valent pneumococcal polysaccharide nontypeable Haemophilus influenzae protein D–conjugated vaccine; TVC, total vaccinated cohort.

**Figure 4.**
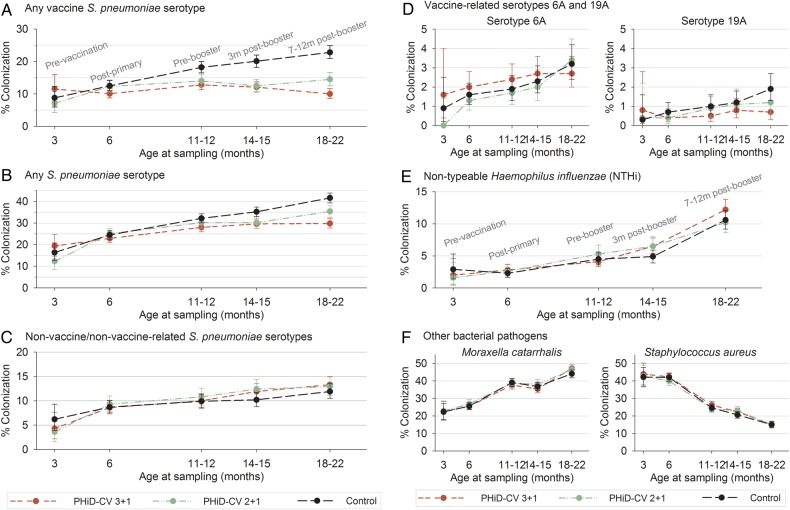
Percentage of infants with nasopharyngeal colonization (infant total vaccinated cohort for carriage). The percentages of infants, enrolled between 6 weeks and 6 months of age, colonized with *S pneumoniae,* NTHi, *M catarrhalis*, or *S aureus* were assessed at different ages: 3 months (before vaccination, only for a subset of infants), 6 months (1 month after primary vaccination), 11–12 months (before booster), 14–15 months (3 months after booster), and 18–22 months (7–12 months after booster). Mean values with 95% confidence intervals are shown. Vaccine-type *S pneumoniae* serotypes were 1, 4, 5, 6B, 7F, 9V, 14, 18C, 19F, and 23F; non-vaccine/non–vaccine-related serotypes were any *S pneumoniae* serotype, excluding vaccine serotypes and excluding serotypes belonging to the same serogroup as vaccine serotypes. Abbreviations: m, months; PHiD-CV, p10-valent pneumococcal polysaccharide nontypeable Haemophilus influenzae protein D–conjugated vaccine.

**Figure 5.**
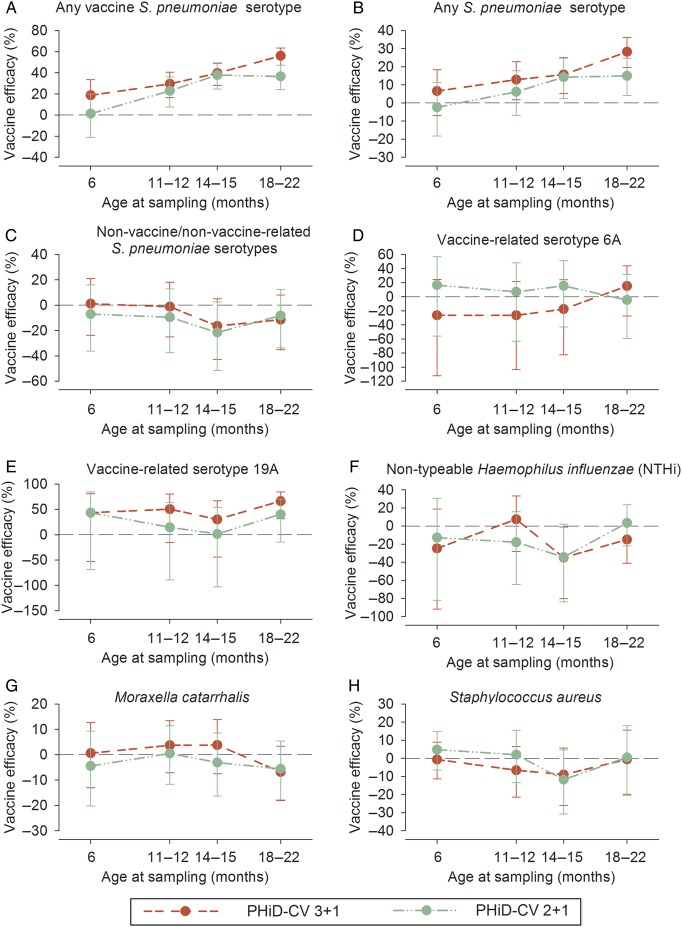
Vaccine effectiveness against nasopharyngeal carriage at given time points (infant total vaccinated cohort for carriage). Vaccine efficacy against nasopharyngeal carriage of *S pneumoniae,* NTHi, *M catarrhalis*, and *S aureus* was assessed at different ages: 6 months (1 month after primary vaccination), 11–12 months (before booster), 14–15 months (3 months after booster), and 18–22 months (7–12 months after booster). Mean values with 95% confidence intervals are shown. Vaccine-type *S pneumoniae* serotypes were 1, 4, 5, 6B, 7F, 9V, 14, 18C, 19F, and 23F; non-vaccine/non–vaccine-related serotypes were any *S pneumoniae* serotype, excluding the vaccine serotypes and any serotype that belonged to the same serogroup as the vaccine serotype. Abbreviation: PHiD-CV, 10-valent pneumococcal polysaccharide nontypeable Haemophilus influenzae protein D–conjugated vaccine.

The most prevalent pneumococcal serotypes in the control group were 6B, 19F, 23F, 6A, and 11A (Figure 3). PHiD-CV vaccination substantially reduced vaccine-serotype carriage. The highest VE were observed following the booster dose: 56.1% at 18–22 months of age in the 3+1 group and 37.9% at 14–15 months of age in the 2+1 group. This carriage reduction was mainly due to decreased carriage of serotypes 6B, 14, 19F, and 23F. With increasing age and the time elapsed after booster vaccination, a trend for increased carriage of non-vaccine/non–vaccine-related serotypes was observed in all the groups, with no major differences between the groups. Altogether, these changes resulted in a net reduction of overall pneumococcal carriage in infants who received pneumococcal vaccination according to either schedule; VE against carriage of all pneumococci increased with age, up to 28.3% and 15.0% for the 3+1 and 2+1 groups, respectively (Figures 4 and 5; Supplementary Table 2). The occurrence of the most common non-vaccine/non–vaccine-related serotypes with a prevalence of >3% is shown in Figure 3B. Of note, prevalence of serotypes 3 and 6C were low (maximum 0.5% and 1.4%, respectively, in the control group).

For vaccine-related serotype 19A, the carriage prevalence was low, with a maximum colonization rate of 3.4% across all visits in the control group (Figure 3). Nevertheless, consistently positive VE against 19A carriage was observed at all post-vaccination time points in the 3+1 group, with a statistically significant VE at the 18- to 22-month time point and across all visits. Point estimates of VE against 19A were in the same range as VE against vaccine-type carriage at the 18- to 22-month time point. For the 2+1 group, a trend for reduction of serotype 19A carriage was observed, but no significant effectiveness was shown at any time point. Although the prevalence of vaccine-related serotype 6A was higher than that of serotype 19A, no consistent effect against 6A carriage was observed for either PHiD-CV schedule (Figure 5; Supplementary Table 2). Trends for VE against cumulative acquisition were similar to those against carriage occurrence at a given time point, with VE against cumulative acquisition of vaccine serotypes ranging across visits from 38.2% to 45.5% for the 3+1 group and from 35.8% to 40.4% for the 2+1 group. VE against cumulative acquisition of *S pneumoniae,* regardless of serotype, ranged from 14.8% to 16.4% for the 3+1 group and from 12.5% to 14.5% for the 2+1 group (Supplementary Table 3). VE against cumulative acquisition from pre- to one month post-primary vaccination, assessed for the immunogenicity subset with pre-vaccination swabs available, is presented in Supplementary Table 4.

#### Catch-Up Groups

A trend toward VE against vaccine-type carriage was observed in the 7- to 11-month and 12- to 18-month catch-up cohorts (Supplementary Tables 5 and 6).

### Effect of PHiD-CV Vaccination on Carriage of Other Bacterial Pathogens

NTHi carriage was low, with 10.6% of infants in the control group colonized at 18–22 months of age. Carriage of *M catarrhalis* or *S aureus* was more common. No differences in carriage of these pathogens were seen between the PHiD-CV and control groups (Figures 4 and 5).

### Effect of PHiD-CV Vaccination on AOM

At least 1 AOM episode was reported for 63.0% (1163 of 1846) of the infants in the 3+1 group, 62.5% (589 of 942) in the 2+1 group, compared to 67.1% (892 of 1329) in the control group. VE in reducing the number of infants for whom ≥1 AOM episode was reported were 6.1% (95% CI, –2.7 to 14.1) for the 3+1 group and 7.4% (95% CI, –2.8 to 16.6) for the 2+1 group. VE in preventing all AOM episodes were 2.8% (95% CI, –9.5 to 13.9) for the 3+1 group and 10.2% (95% CI, –4.1 to 22.9) for the 2+1 schedule (Table 1). VE for both schedules combined were 6.7% (95% CI, –1.3 to 14.0) for reducing the number of infants for whom ≥1 AOM episode was reported and 6.4% (95% CI, –5.5 to 17.2) for preventing all AOM episodes (Table 2).

**Table 1.** Vaccine Effectiveness Against Acute Otitis Media (Infant TVC for AOM Analysis)

AOM Episodes	Infant PHiD-CV						Infant Controls (N = 1329; FU = 2012)	
	3+1 (N = 1846; FU = 2765)			2+1 (N = 942; FU = 1417)				
	n	n/FU	VE (% [95% CI])	n	n/FU	VE (% [95% CI])	n	n/FU
≥1	1163	421	6.1 (–2.7 to 14.1)	589	416	7.4 (–2.8 to 16.6)	892	443
≥1, with antibiotics	1133	410	6.1 (–2.8 to 14.2)	579	409	6.4 (–4.0 to 15.8)	867	431
All	2753	996	2.8 (–9.5 to 13.9)	1375	970	10.2 (–4.1 to 22.9)	2033	1011
All, with antibiotics	2662	963	2.0 (–11.3 to 13.8)	1322	933	10.8 (–5.5 to 24.7)	1964	976

Analysis was performed on the total vaccinated cohort for acute otitis media effectiveness.

Abbreviations: AOM, acute otitis media; CI, confidence interval; FU, sum of follow-up periods, expressed in years; N, total number of children in the specified cohort; n, number of children or episodes; n/FU, incidence of children with ≥1 AOM episode or incidence of all AOM episodes, expressed in 1000 child-years; PHiD-CV, 10-valent pneumococcal polysaccharide nontypeable *Haemophilus influenzae* protein D–conjugated vaccine; TVC, total vaccinated cohort; VE, vaccine effectiveness.

The vast majority (>97%) of the infants with AOM received antimicrobial treatment. VE against AOM with antimicrobial prescription was in line with the corresponding overall effectiveness against AOM (Table 2).

**Table 2.** Vaccine Effectiveness Against Acute Otitis Media for both Schedules Combined

AOM Episodes	Infant PHiD-CV (N = 2788; FU = 4182)			Infant Controls (N = 1329; FU = 2012)	
	n	n/FU	VE (% [95% CI])	n	n/FU
≥1	1752	419	6.7 (–1.3 to 14.0)	892	443
All	4128	987	6.4 (–5.5 to 17.2)	2033	1011

Analysis was performed on the total vaccinated cohort for acute otitis media effectiveness.

Abbreviations: AOM, acute otitis media; CI, confidence interval; FU, sum of follow-up periods, expressed in years; N, total number of children in the specified cohort; n, number of children or episodes; n/FU, incidence of children with ≥1 AOM episode or incidence of all AOM episodes, expressed in 1000 child-years; PHiD-CV, 10-valent pneumococcal polysaccharide nontypeable *Haemophilus influenzae* protein D–conjugated vaccine; VE, vaccine effectiveness.

A post-hoc analysis comparing pre- and post-booster effectiveness suggested higher effectiveness post-booster in reducing the number of infants reporting ≥1 AOM episode, while VE against all episodes seemed to be lower post-booster (Table 3).

**Table 3.** Vaccine Effectiveness Against Acute Otitis Media Before and After Booster Vaccination

AOM Episodes	Infant PHiD-CV						Infant Controls (N = 1291; FU = 1992)	
	3+1 (N = 1783; FU = 2735)			2+1 (N = 917; FU = 1406)				
	n	n/FU	VE (% [95% CI])	n	n/FU	VE (% [95% CI])	n	n/FU
≥1, before booster	628	230	4.5 (–18.2 to 22.6)	327	233	7.9 (–13.6 to 26.0)	485	244
≥1, after booster	423	155	8.6 (–5.7 to 20.9)	216	154	12.0 (–7.1 to 28.2)	347	174
All, before booster	1082	396	4.5 (–9.7 to 16.6)	549	390	10.2 (–8.1 to 26.1)	812	408
All, after booster	1640	560	1.9 (–11.6 to 14.3)	824	586	8.2 (–5.6 to 20.9)	1205	605

Analysis was performed on the total vaccinated cohort for acute otitis media analysis considering only infants with the full vaccination schedule (4 doses for the 3+1 or 3 doses for the 2+1 schedule).

Abbreviations: AOM, acute otitis media; CI, confidence interval; FU, sum of follow-up periods, expressed in years; N, total number of children in the specified cohort; n, number of children or episodes; n/FU, incidence of children with ≥1 AOM episode or incidence of all AOM episodes, expressed in 1000 child-years; PHiD-CV, 10-valent pneumococcal polysaccharide nontypeable *Haemophilus influenzae* protein D–conjugated vaccine; VE, vaccine effectiveness.

### Immunogenicity

Post-primay vaccination, for each of the vaccine serotypes, ≥79.3% of the infants who received the 3+1 schedule and ≥66.3% of those who received the 2+1 schedule had antibody concentrations of ≥0.2 µg/mL. Post-booster, these percentages were ≥94.7% and ≥96.9% for the 3+1 and 2+1 groups, respectively. Antibody geometric mean concentrations (GMCs) and opsonophagocytic assay (OPA) geometric mean titers (GMTs) were higher post-booster than post-primary vaccination, except for serotype 6B GMTs in the PHiD-CV 3+1 group, which remained in the same range. Antibody GMCs and OPA GMTs tended to be lower in the 2+1 group than in the 3+1 group for most serotypes, especially post-primary vaccination (Supplementary Tables 7 and 8).

For each of the vaccine serotypes, the percentages of children with antibody concentrations of ≥0.2 µg/mL were ≥60.3% in the 7- to 11-month catch-up group and ≥86.2% in the 12- to 18-month catch-up group 1 month after dose 2 and ≥90.5% for the 7- to 11-month catch-up group post-booster. In the 7- to 11-month group, higher GMCs were observed post-booster than those post-primary vaccination, except the GMCs for serotype 4, which remained in the same range (Supplementary Table 7).

### Safety and Reactogenicity

The PHiD-CV was well-tolerated and showed an acceptable safety profile (Supplementary Figure 1; Supplementary Table 9). Reactogenicity was expected to be higher for PHiD-CV than the control vaccines because of the known low reactogenicity of the hepatitis vaccine. Dose 2 of the infant 3+1 schedule was given without concomitant vaccinations and thus illustrates the reactogenicity of PHiD-CV vaccination alone.

Serious adverse events considered by the investigator to be causally related to vaccination were reported for 4 infants in the PHiD-CV 3+1 group (sepsis with non-specified etiology in 1 infant, pyrexia in 1 infant, and convulsion in 2 infants), for none in the PHiD-CV 2+1 group, for 2 in the infant control groups (petit mal epilepsy in 1 infant and pyrexia in 1 infant), and for none in the catch-up groups (Supplementary Table 9). One fatal serious adverse event (sudden infant death, not considered vaccination related) was reported in the infant PHiD-CV 2+1 group.

## DISCUSSION

In this cluster-randomized study, nasopharyngeal carriage of vaccine-type pneumococci and their acquisition was reduced after PHiD-CV vaccination. Effectiveness against vaccine-type pneumococcal carriage was observed with both the 3+1 and 2+1 infant schedules, although no VE was observed 1 month after the primary vaccination (infant age 6 months) for the 2+1 schedule. VE for the 3+1 and 2+1 schedules were at similar ranges 6 months post-primary vaccination (age 11–12 months) and 3 months post-booster (age 14–15 months). At the 18- to 22-month time point, VE continued to increase only for those who received the 3+1 schedule and tended to be higher than for those who received the 2+1 schedule. Our results suggest that PHiD-CV vaccination of children may induce herd protection against vaccine-type disease. This hypothesis can be supported by indirect carriage effects observed in FinIP [19], reports showing a decline in vaccine-type carriage [20] and vaccine-type IPD [21–24] across all age groups after PHiD-CV vaccination of children, and preliminary data showing decreases in pneumonia in unvaccinated children not eligible for the national vaccination program [25].

The reduction in vaccine-type carriage was mainly a result of decreased carriage of the most prevalent serotypes, 6B, 14, 19F, and 23F. Post-vaccination, antibody levels against serotypes 6B and 23F were low, consistent with previous reports [10, 13]. Nevertheless, we observed reduced carriage of both serotypes. Furthermore, 100% PHiD-CV effectiveness against serotype 6B IPD has been reported [14], but the antibody levels needed to confer protection against IPD may be lower than those for nasopharyngeal carriage.

We also noted reduced carriage of vaccine-related serotype 19A with the 3+1 PHiD-CV schedule, although the carriage rates were low and, thus, the CIs were large. This finding may fit with observations after PHiD-CV infant immunization showing decreased serotype 19A IPD [26–29]. We observed positive trends but no significant effectiveness against 19A colonization in the 2+1 group.

No consistent effect on the carriage of vaccine-related serotype 6A was observed for the 2+1 schedule or for the 3+1 PHiD-CV schedule, although this serotype had a higher prevalence than 19A, and a reduction of 6A IPD after introduction of the PHiD-CV into the Finnish NVP was reported recently [29]. Similarly, some of the early PCV7 trials did not find a clear impact of vaccination on 6A carriage [30], whereas dramatically decreased 6A carriage was observed after widespread adoption of PCV7 [31], in addition to significant herd protection against serotype 6A IPD [32, 33].

Increases in non–vaccine-serotype carriage were limited and observed only at later study visits. Similar trends were noted in previous PHiD-CV studies [34, 35]. In contrast, PCV7 studies showed early and pronounced replacement [36]. Although the degree of replacement in nasopharyngeal carriage may be relative to the degree of vaccine-type reduction, replacement is also affected by changes in the entire microbiome, which, in addition to being under vaccine pressure, are also affected by viral coinfections, antibiotic selection of serotypes commonly resistant to antibiotics, clonal mutants quickly spreading, secular trends in serotype prevalence, etc.

We found no impact of PHiD-CV vaccination on NTHi carriage, which is consistent with previous PHiD-CV reports [37, 38]. The 11-valent PHiD-CV predecessor vaccine had a 38.6% (95% CI, –6.3 to 64.6) reduction in NTHi carriage 3 months after a booster, but the difference between groups had disappeared by 12 months post-booster [35].

No significant effectiveness in reducing AOM rates was observed for the 3+1 or 2+1 regimen. Nevertheless, a low but consistent trend for effectiveness in reducing AOM was observed for each PHiD-CV vaccination regimen. No major differences between the 3+1 and 2+1 schedules were seen; however, this study was not designed to detect schedule differences. PHiD-CV VE against hospital-treated pneumonia [39] and against outpatient purchases of antimicrobial drugs [15] were similar for both schedules.

A possible limitation is the collection of information about physician-diagnosed AOM from the parents. Nevertheless, Finland has well-established diagnostic and management guidelines for AOM [18], and the observed incidences were similar to those reported in a Finnish PCV7 efficacy study [5]. Moreover, most participants with AOM received an antimicrobial prescription (>97%), which is in line with national guidelines that recommend antibiotic treatment when an AOM diagnosis is certain [18]. The AOM study end point could be regarded as an antimicrobial-treated AOM end point.

Last, because this study was part of a cluster-randomized study, the observed effectiveness against carriage and AOM could be higher than the vaccine's efficacy as a result of herd protection within the vaccinated clusters. However, vaccination uptake rates per cluster were low to moderate (8%–61%) [14], and carriage assessment was completed within 2 years after start of the study, which thus limits the possibility of observing a herd effect on the vaccine recipients.

PHiD-CV administered according to different age-appropriate schedules resulted in an acceptable safety and immunogenicity profile. Antibody GMCs and OPA GMTs were higher after the 3-dose than the 2-dose primary schedule; these differences diminished post-booster vaccination.

## CONCLUSIONS

The observed effectiveness against nasopharyngeal carriage of vaccine-type pneumococci indicates the potential of PHiD-CV to induce a direct effect and herd protection against vaccine-type pneumococcal disease. Only a limited increase in the carriage of non–vaccine-type pneumococcal serotypes was seen at later time points, which resulted in overall decreased carriage of all pneumococci.

After infant PHiD-CV vaccination, we noted a trend toward decreased numbers of parent-reported physician-diagnosed AOM episodes. PHiD-CV had an acceptable safety profile.

## Supplementary Material

Supplementary Data

## Supplementary Data

Supplementary materials are available at the *Journal of The Pediatric Infectious Diseases Society* online (http://jpids.oxfordjournals.org). Supplementary materials consist of data provided by the author that are published to benefit the reader. The posted materials are not copyedited. The contents of all supplementary data are the sole responsibility of the authors. Questions or messages regarding errors should be addressed to the author.
