# Supplementary material for: Effectiveness of the 10-Valent Pneumococcal Nontypeable Haemophilus influenzae Protein D–Conjugated Vaccine (PHiD-CV) Against Carriage and Acute Otitis Media—A Double-Blind Randomized Clinical Trial in Finland
Source: J Pediatric Infect Dis Soc. 2016 Apr 28;5(3):237–48. doi: 10.1093/jpids/piw010 (PMC5125453; doi:10.1093/jpids/piw010)
Supplement: Supplementary Data [file supp_5_3_237__index.html]

Effectiveness of the 10-Valent Pneumococcal Nontypeable Haemophilus influenzae Protein D–Conjugated Vaccine (PHiD-CV) Against Carriage and Acute Otitis Media—A Double-Blind Randomized Clinical Trial in Finland — Supplementary Data 

# Effectiveness of the 10-Valent Pneumococcal Nontypeable *Haemophilus influenzae* Protein D–Conjugated Vaccine (PHiD-CV) Against Carriage and Acute Otitis Media—A Double-Blind Randomized Clinical Trial in Finland

## Supplementary Data

Supplementary Data

- Supplementary Data - Docx file
