# Supplementary material for: Effectiveness of the 10-Valent Pneumococcal Nontypeable Haemophilus influenzae Protein D–Conjugated Vaccine (PHiD-CV) Against Carriage and Acute Otitis Media—A Double-Blind Randomized Clinical Trial in Finland
Source: J Pediatric Infect Dis Soc. 2016 Apr 28;5(3):237–48. doi: 10.1093/jpids/piw010 (PMC5125453; doi:10.1093/jpids/piw010)
Supplement: Supplementary Data [file supp_piw010_piw010supp.docx]

**Supplementary Figure 1. Solicited local and general symptoms (Infant TVC for safety)**

*Reactogenicity overall for primary doses, and after the booster dose:*

Note that reactogenicity was expected to be higher for PHiD-CV than control vaccines, because hepatitis vaccines are known to have a low reactogenicity profile. Routine pediatric vaccines such as DTPa IPV/Hib and human rotavirus vaccine were co-administered at 3 and 5 months of age; DTPa IPV/Hib was also co-administered at 11–12 months. N, number of children in the specified group.

*Reactogenicity post-dose 2:*

Dose 2 of the infant 3+1 schedule was given without concomitant vaccinations and thus illustrates the reactogenicity of PHiD-CV vaccination alone. TVC, total vaccinated cohort.

**Supplementary Table 1. Demographic characteristics**

| A. TVC for carriage/safety | Infant cohort | | | | | | |  | Catch-up cohort | | | | |
| --- | --- | --- | --- | --- | --- | --- | --- | --- | --- | --- | --- | --- | --- |
|  | (6W–6M) | | | | | | |  | 7–11M catch-up group | |  | 12–18M catch-up group | |
|  | PHiD-CV 3+1  N=1849 | | Control  3+1  N=1069 | | PHiD-CV 2+1  N=1316 | | Control  2+1  N=859 |  | PHiD-CV  N=241 | Control  N=204 |  | PHiD-CV  N=368 | Control  N=271 |
| Age (months) ± SD: |  | |  | |  | |  |  |  |  |  |  |  |
| Mean age at dose 1 | 2.4 ± 1.02 | | 2.6 ± 1.19 | | 2.3 ± 0.95 | | 2.4 ± 1.00 |  | 9.0 (1.44) | 8.7 (1.39) |  | 15.0 (1.99) | 15.2 (1.99) |
| Mean age at dose 2 | 3.5 ± 1.07 | | 3.7 ± 1.24 | | 4.4 ± 1.01 | | 4.5 ± 1.09 |  | 10.1 (1.50) | 9.9 (1.51) |  | 21.5 (2.18) | 21.7 (2.15) |
| Mean age at dose 3 | 4.7 ± 1.12 | | 4.9 ± 1.27 | | 11.2 ± 0.55 | | 11.2 ± 0.69 |  | 15.8 (1.68) | 15.7 (1.76) |  | - | - |
| Mean age at dose 4 | 11.2 ± 0.75 | | 11.3 ± 0.76 | | - | | - |  | - | - |  | - | - |
| Gender: n (%) female | 921 (49.8) | | 551 (51.5) | | 681 (51.7) | | 393 (45.8) |  | 118 (49.0) | 113 (55.4) |  | 173 (47.0) | 142 (52.4) |
| Ethnicity: n (%) |  | |  | |  | |  |  |  |  |  |  |  |
| Caucasian | 1822 (98.5) | | 1058 (99.0) | | 1303 (99.0) | | 845 (98.4) |  | 235 (97.5) | 201 (98.5) |  | 362 (98.4) | 270 (99.6) |
| Other | 27 (1.5) | | 11 (1.0) | | 13 (1.0) | | 14 (1.6) |  | 6 (2.5) | 3 (1.5) |  | 6 (1.6) | 1 (0.4) |
| B. TVC for AOM effectiveness | | | | | | |  |  |  |  |  |  |  |
|  | | Infant cohort  (6W–6M) | | | | |  |  |  |  |  |  |  |
|  | | PHiD-CV 3+1  N=1846 | | PHiD-CV 2+1  N=942 | | Control  N=1329 |  |  |  |  |  |  |  |
| Age (months) ± SD: | |  | |  | |  |  |  |  |  |  |  |  |
| Mean age at dose 1 | | 2.4 ± 1.03 | | 2.2 ± 0.74 | | 2.3 ± 0.95 |  |  |  |  |  |  |  |
| Gender: n (%) female | | 920 (49.8) | | 484 (51.4) | | 644 (48.5) |  |  |  |  |  |  |  |
| Ethnicity: n (%) | |  | |  | |  |  |  |  |  |  |  |  |
| Caucasian | | 1819 (98.5) | | 930 (98.7) | | 1310 (98.6) |  |  |  |  |  |  |  |
| Other | | 27 (1.5) | | 12 (1.3) | | 19 (1.4) |  |  |  |  |  |  |  |
| Mean duration of follow-up (months): | | 18.0 | | 18.1 | | 18.2 |  |  |  |  |  |  |  |

TVC, total vaccinated cohort; AOM, acute otitis media; W, weeks; M, months; SD, standard deviation; N, number of children in the specified group; n, number of children with the specified characteristic.

**Supplementary Table 2. Nasopharyngeal carriage at a given timepoint, and vaccine effectiveness per visit and overall; infant groups (TVC for carriage)**

|  | **Infant control**  N*=1897 | | **Infant PHiD-CV 3+1**  N*=1803 | | | **Infant PHiD-CV 2+1**  N*=1289 | | | |
| --- | --- | --- | --- | --- | --- | --- | --- | --- | --- |
|  |  | **Colonization** |  | **Colonization** | **Vaccine effectiveness** |  | | **Colonization** | **Vaccine effectiveness** |
|  | n | % (95% CI) | n | % (95% CI) | % (95% CI) | n | | % (95% CI) | % (95% CI) |
| ***Streptococcus pneumoniae*** | | | | | | | | | |
| **Any vaccine serotype** | | | | | | | | | |
| 3M* | 30 | 8.8 (6.0; 12.3) | 29 | 11.5 (7.8; 16) | - | 18 | 7.1 (4.3; 11.0) | | - |
| 6M | 237 | 12.5 (11.0; 14.1) | 183 | 10.1 (8.8; 11.6) | 18.8 (1.1; 33.4) | 159 | 12.3 (10.6; 14.3) | | 1.3 (-21.2; 19.8) |
| 11–12M | 342 | 18.2 (16.5; 20.0) | 229 | 12.8 (11.3; 14.5) | 29.6 (16.5; 40.7) | 178 | 14.0 (12.2; 16.1) | | 23.0 (7.5; 36.1) |
| 14–15M | 364 | 20.1 (18.2; 22.0) | 209 | 12.1 (10.6; 13.7) | 39.7 (28.3; 49.4) | 153 | 12.5 (10.7; 14.4) | | 37.9 (24.7; 48.9) |
| 18–22M | 404 | 22.8 (20.9; 24.9) | 169 | 10.0 (8.6; 11.6) | 56.1 (47.3; 63.5) | 176 | 14.5 (12.5; 16.6) | | 36.6 (24.2; 47.2) |
| *Across all visits* | *802* | *42.0 (39.8; 44.2)* | *476* | *26.2 (24.2; 28.3)* | *37.6 (30.0; 44.4)* | *390* | *30.1 (27.6; 32.6)* | | *28.4 (19.1; 36.7)* |
| **Any serotype** | | | | | | | | | |
| 3M* | 56 | 16.4 (12.7; 20.8) | 49 | 19.4 (14.7; 24.8) | - | 31 | 12.3 (8.5; 16.9) | | - |
| 6M | 464 | 24.5 (22.5; 26.5) | 412 | 22.9 (20.9; 24.9) | 6.6 (-6.9; 18.4) | 323 | 25.1 (22.7; 27.5) | | -2.4 (-18.3; 11.4) |
| 11–12M | 604 | 32.2 (30.1; 34.3) | 500 | 28.0 (26.0; 30.2) | 12.9 (1.8; 22.8) | 383 | 30.2 (27.7; 32.8) | | 6.2 (-6.8; 17.7) |
| 14–15M | 638 | 35.2 (33.0; 37.4) | 512 | 29.6 (27.5; 31.9) | 15.7 (5.2; 25.1) | 370 | 30.2 (27.6; 32.8) | | 14.3 (2.4; 24.8) |
| 18–22M | 736 | 41.6 (39.3; 43.9) | 503 | 29.8 (27.7; 32.1) | 28.3 (19.6; 36.1) | 430 | 35.4 (32.7; 38.1) | | 15.0 (4.1; 24.7) |
| *Across all visits* | *1237* | *64.8 (62.6; 66.9)* | *1041* | *57.3 (55.0; 59.6)* | *11.5 (3.8; 18.6)* | *774* | *59.7 (56.9; 62.4)* | | *7.9 (-0.9; 15.9)* |
| **Non-vaccine/non-vaccine-related serotypes** | | | | | | | | | |
| 3M* | 21 | 6.2 (3.9; 9.3) | 11 | 4.3 (2.2; 7.6) | - | 9 | 3.6 (1.6; 6.6) | | - |
| 6M | 165 | 8.7 (7.5; 10.1) | 155 | 8.6 (7.3; 10.0) | 1.2 (-23.8; 21.1) | 120 | 9.3 (7.8; 11.0) | | -7.0 (-36.2; 16.1) |
| 11–12M | 185 | 9.9 (8.5; 11.3) | 178 | 10.0 (8.6; 11.5) | -1.2 (-25.0; 18.1) | 137 | 10.8 (9.1; 12.6) | | -9.5 (-37.4; 12.8) |
| 14–15M | 185 | 10.2 (8.8; 11.7) | 205 | 11.9 (10.4; 13.5) | -16.4 (-42.8; 5.1) | 152 | 12.4 (10.6; 14.4) | | -21.5 (-51.4; 2.6) |
| 18–22M | 211 | 11.9 (10.5; 13.5) | 224 | 13.3 (11.7; 15.0) | -11.4 (-35.1; 8.1) | 157 | 12.9 (11.1; 14.9) | | -8.2 (-33.7; 12.5) |
| *Across all visits* | *517* | *27.1 (25.1; 29.1)* | *528* | *29.1 (27.0; 31.2)* | *-7.4 (-21.5; 5.0)* | *393* | *30.3 (27.8; 32.9)* | | *-11.9 (-27.9; 2.1)* |
| **Vaccine-related serotype 6A** | | | | | | | | | |
| 3M* | 3 | 0.9 (0.2; 2.5) | 4 | 1.6 (0.4; 4.0) | - | 0 | 0.0 (0.0; 1.4) | | - |
| 6M | 30 | 1.6 (1.1; 2.2) | 36 | 2.0 (1.4; 2.8) | -26.3 (-112.2; 24.4) | 17 | 1.3 (0.8; 2.1) | | 16.6 (-56.1; 56.9) |
| 11–12M | 35 | 1.9 (1.3; 2.6) | 42 | 2.4 (1.7; 3.2) | -26.3 (-103.7; 21.3) | 22 | 1.7 (1.1; 2.6) | | 7.0 (-63.0; 48.0) |
| 14–15M | 42 | 2.3 (1.7; 3.1) | 47 | 2.7 (2.0; 3.6) | -17.5 (-82.6; 24.1) | 24 | 2.0 (1.3; 2.9) | | 15.5 (-42.8; 51.1) |
| 18–22M | 57 | 3.2 (2.4; 4.2) | 46 | 2.7 (2.0; 3.6) | 15.3 (–27.1; 43.9) | 41 | 3.4 (2.4; 4.5) | | -4.6 (-59.1; 31.7) |
| *Across all visits* | *119* | *6.2 (5.2; 7.4)* | *121* | *6.7 (5.6; 7.9)* | *-6.9 (-38.9; 17.7)* | *78* | *6.0 (4.8; 7.4)* | | *3.5 (-29.5; 28.4)* |
| **Vaccine-related serotype 19A** | | | | | | | | | |
| 3M* | 1 | 0.3 (0.0; 1.6) | 2 | 0.8 (0.1; 2.8) | - | 1 | 0.4 (0.0; 2.2) | | - |
| 6M | 13 | 0.7 (0.4; 1.2) | 7 | 0.4 (0.2; 0.8) | 43.3 (-52.8; 80.9) | 5 | 0.4 (0.1; 0.9) | | 43.4 (-69.2; 84.2) |
| 11–12M | 19 | 1.0 (0.6; 1.6) | 9 | 0.5 (0.2; 1.0) | 50.2 (-15.6; 80.1) | 11 | 0.9 (0.4; 1.5) | | 14.4 (-89.4; 63.2) |
| 14–15M | 21 | 1.2 (0.7; 1.8) | 14 | 0.8 (0.4; 1.4) | 30.0 (-44.4; 67.1) | 14 | 1.1 (0.6; 1.9) | | 1.4 (-103.2; 53.6) |
| 18–22M | 34 | 1.9 (1.3; 2.7) | 11 | 0.7 (0.3; 1.2) | 66.1 (31.4; 84.5) | 14 | 1.2 (0.6; 1.9) | | 40.1 (-14.6; 70.3) |
| *Across all visits* | *64* | *3.4 (2.6; 4.3)* | *32* | *1.8 (1.2; 2.5)* | *47.4 (18.4; 66.7)* | *38* | *2.9 (2.1; 4.0)* | | *12.6 (-32.6; 43.1)* |
| **NTHi** | | | | | | | | | |
| 3M* | 10 | 2.9 (1.4; 5.3) | 5 | 2.0 (0.6; 4.6) | - | 4 | 1.6 (0.4; 4.0) | | - |
| 6M | 43 | 2.3 (1.6; 3.0) | 51 | 2.8 (2.1; 3.7) | -24.8 (-91.8; 18.5) | 33 | 2.6 (1.8; 3.6) | | -12.9 (-82.0; 30.5) |
| 11–12M | 84 | 4.5 (3.6; 5.5) | 74 | 4.1 (3.3; 5.2) | 7.3 (-28.2; 33.1) | 67 | 5.3 (4.1; 6.7) | | -18.0 (-64.6; 15.7) |
| 14–15M | 88 | 4.9 (3.9; 5.9) | 113 | 6.5 (5.4; 7.8) | -35.0 (-80.4; -1.2) | 80 | 6.5 (5.2; 8.0) | | -34.4 (-84.0; 2.0) |
| 18–22M | 187 | 10.6 (9.2; 12.1) | 205 | 12.2 (10.6; 13.8) | -15.1 (-41.1; 6.1) | 124 | 10.2 (8.6; 12.1) | | 3.3 (-22.0; 23.5) |
| *Across all visits* | *363* | *19.0 (17.3; 20.8)* | *391* | *21.5 (19.7; 23.5)* | *-13.3 (-31.0; 2.0)* | *264* | *20.4 (18.2; 22.7)* | | *-7.1 (-25.8; 9.0)* |
| ***Moraxella catarrhalis*** | | | | | | | | | |
| 3M* | 76 | 22.3 (18.0; 27.1) | 57 | 22.5 (17.5; 28.2) | - | 58 | 22.9 (17.9; 28.6) | | - |
| 6M | 486 | 25.6 (23.7; 27.6) | 459 | 25.5 (23.5 ;27.5) | 0.6 (-13.1; 12.7) | 345 | 26.8 (24.4; 29.3) | | -4.5 (-20.2; 9.3) |
| 11–12M | 733 | 39.1 (36.8; 41.3) | 671 | 37.6 (35.4; 39.9) | 3.7 (-7.1; 13.4) | 493 | 38.8 (36.2; 41.6) | | 0.5 (-11.7; 11.4) |
| 14–15M | 668 | 36.8 (34.6; 39.1) | 612 | 35.4 (33.2; 37.7) | 3.8 (-7.5; 13.9) | 466 | 38.0 (35.3; 40.8) | | -3.1 (-16.3; 8.6) |
| 18–22M | 780 | 44.1 (41.8; 46.4) | 794 | 47.1 (44.7; 49.5) | -6.8 (-18.1; 3.4) | 566 | 46.5 (43.7; 49.4) | | -5.6 (-17.8; 5.4) |
| *Across all visits* | *1470* | *77.0 (75.0; 78.8)* | *1403* | *77.3 (75.3; 79.2)* | *-0.4 (-8.1; 6.8)* | *1014* | *78.2 (75.8; 80.4)* | | *-1.6 (-10.1; 6.3)* |
| ***Staphylococcus aureus*** | | | | | | | | | |
| 3M* | 144 | 42.2 (36.9; 47.7) | 111 | 43.9 (37.7; 50.2) | - | 108 | 42.7 (36.5; 49.0) | | - |
| 6M | 796 | 42.0 (39.7; 44.2) | 762 | 42.3 (40.0; 44.6) | -0.7 (-11.4; 8.9) | 515 | 40.0 (37.3; 42.7) | | 4.8 (-6.5; 14.9) |
| 11–12M | 462 | 24.6 (22.7; 26.6) | 468 | 26.2 (24.2; 28.3) | -6.6 (-21.5; 6.5) | 306 | 24.1 (21.8; 26.6) | | 2.0 (-13.4; 15.5) |
| 14–15M | 373 | 20.6 (18.7; 22.5) | 387 | 22.4 (20.5; 24.5) | -9.0 (-26.0; 5.7) | 282 | 23.0 (20.7; 25.4) | | -11.8 (-30.8; 4.6) |
| 18–22M | 266 | 15.0 (13.4; 16.8) | 255 | 15.1 (13.4; 16.9) | -0.6 (-19.9; 15.6) | 182 | 15.0 (13.0; 17.1) | | 0.5 (-20.6; 18.0) |
| *Across all visits* | *1222* | *64.0 (61.8; 66.1)* | *1190* | *65.5 (63.3; 67.7)* | *-2.4 (-11.0; 5.5)* | *838* | *64.6 (61.9; 67.2)* | | *-1.0 (-10.4; 7.6)* |

N, maximum number of cultured swabs per visit; n/%, number/percentage of children with swabs associated to the specified serotype/bacteria at the considered visit (or after at least 1 visit for “across all visits”); M, months; TVC, total vaccinated cohort; CI, confidence interval; NTHi, non-typeable *Haemophilus influenzae*.
* 3M: pre-vaccination (only for the immunogenicity subset of infants, with N=341 for control, N=253 for PHiD-CV 3+1, and N=253 for PHiD-CV 2+1)
6M: 1 month post-primary vaccination; 11–12M: pre-booster; 14–15M: 3 months post-booster; 18–22M: 7–12M post-booster.

**Supplementary Table 3. Cumulative acquisition of nasopharyngeal carriage and vaccine effectiveness for infants enrolled between 6 weeks and 6 months of age, from 6 months onwards (Infant TVC for carriage)**

|  | **Infant control**  N=1874 | | **Infant PHiD-CV 3+1**  N=1780 | | | **Infant PHiD-CV 2+1**  N=1269 | | | |
| --- | --- | --- | --- | --- | --- | --- | --- | --- | --- |
|  |  | **Cumulative acquisition rate** |  | **Cumulative acquisition rate** | **Vaccine effectiveness** |  | | **Cumulative acquisition rate** | **Vaccine effectiveness** |
|  | n | % (95% CI) | n | % (95% CI) | % (95% CI) | n | | % (95% CI) | % (95% CI) |
| ***Streptococcus pneumoniae*** | | | | | | | | | |
| **Any vaccine serotype** | | | | | | | | | |
| 11–12M | 223 | 11.9 (10.5; 13.5) | 131 | 7.4 (6.2; 8.7) | 38.2 (22.9; 50.5) | 97 | 7.6 (6.2; 9.2) | | 35.8 (18.1; 49.9) |
| 14–15M | 387 | 21.4 (19.5; 23.4) | 221 | 12.8 (11.3; 14.5) | 40.1 (29.2; 49.5) | 156 | 12.8 (10.9; 14.8) | | 40.4 (28.0; 50.8) |
| 18–22M | 626 | 35.7 (33.5; 38.0) | 326 | 19.5 (17.6; 21.4) | 45.5 (37.6; 52.5) | 269 | 22.4 (20.1; 24.9) | | 37.3 (27.5; 45.8) |
|  |  |  |  |  |  |  |  | |  |
| **Any serotype** | | | | | | | | | |
| 11–12M | 415 | 22.1 (20.3; 24.1) | 331 | 18.6 (16.8; 20.5) | 16.0 (2.7; 27.5) | 246 | 19.4 (17.2; 21.7) | | 12.5 (-2.7; 25.5) |
| 14–15M | 692 | 38.3 (36.0; 40.6) | 562 | 32.6 (30.4; 34.9) | 14.8 (4.7; 23.9) | 400 | 32.7 (30.1; 35.4) | | 14.5 (3.2; 24.6) |
| 18–22M | 1023 | 58.4 (56.0; 60.7) | 818 | 48.8 (46.4; 51.3) | 16.4 (8.2; 23.8) | 609 | 50.8 (47.9; 53.6) | | 13.1 (3.8; 21.5) |
| **Non-vaccine/non-vaccine-related serotypes** | | | | | | | | | |
| 11–12M | 139 | 7.4 (6.3; 8.7) | 135 | 7.6 (6.4; 8.9) | -2.3 (-30.5; 19.9) | 106 | 8.4 (6.9; 10.0) | | -12.6 (-46.0; 13.4) |
| 14–15M | 245 | 13.6 (12.0; 15.2) | 253 | 14.7 (13.0; 16.4) | -8.3 (-29.6; 9.5) | 189 | 15.5 (13.5; 17.6) | | -14.1 (-38.5; 6.1) |
| 18–22M | 381 | 21.7 (19.8; 23.8) | 412 | 24.6 (22.6; 26.7) | -13.1 (-30.4; 1.8) | 295 | 24.6 (22.2; 27.1) | | -13.0 (-31.9; 3.2) |
| **Vaccine-related serotype 6A** | | | | | | | | | |
| 11–12M | 22 | 1.2 (0.7; 1.8) | 28 | 1.6 (1.0; 2.3) | -34.0 (-145.8; 26.1) | 14 | 1.1 (0.6; 1.8) | | 6.0 (-92.1; 55.5) |
| 14–15M | 47 | 2.6 (1.9; 3.4) | 51 | 3.0 (2.2; 3.9) | -13.8 (-72.9; 24.9) | 26 | 2.1 (1.4; 3.1) | | 18.2 (-34.8; 51.4) |
| 18–22M | 90 | 5.1 (4.2; 6.3) | 86 | 5.1 (4.1; 6.3) | 0.1 (-35.8; 26.5) | 61 | 5.1 (3.9; 6.5) | | 1.0 (-38.5; 29.7) |
| **Vaccine-related serotype 19A** | | | | | | | | | |
| 11–12M | 12 | 0.6 (0.3; 1.1) | 6 | 0.3 (0.1; 0.7) | 47.4 (-51.5; 83.8) | 9 | 0.7 (0.3; 1.3) | | -10.8 (-186.4; 58.8) |
| 14–15M | 26 | 1.4 (0.9; 2.1) | 15 | 0.9 (0.5; 1.4) | 39.5 (-18.6; 70.2) | 19 | 1.6 (0.9; 2.4) | | -8.1 (-102.9; 43.5) |
| 18–22M | 51 | 2.9 (2.2; 3.8) | 24 | 1.4 (0.9; 2.1) | 50.8 (18.6; 71.0) | 32 | 2.7 (1.8; 3.7) | | 8.4 (-45.3; 43.0) |
| **NTHi** | | | | | | | | | |
| 11–12M | 80 | 4.3 (3.4; 5.3) | 68 | 3.8 (3.0; 4.8) | 10.5 (-25.2; 36.2) | 60 | 4.7 (3.6; 6.0) | | -10.8 (-56.7; 22.1) |
| 14–15M | 150 | 8.3 (7.1; 9.7) | 164 | 9.5 (8.2; 11.0) | -14.7 (-44.1; 8.6) | 130 | 10.6 (9.0; 12.5) | | -28.2 (-63.2; -0.5) |
| 18–22M | 306 | 17.5 (15.7; 19.3) | 335 | 20.0 (18.1; 22.0) | -14.6 (-34.3; 2.1) | 229 | 19.1 (17.0; 21.5) | | -9.6 (-30.5; 8.1) |
| ***Moraxella catarrhalis*** | | | | | | | | | |
| 11–12M | 481 | 25.7 (23.7; 27.7) | 451 | 25.3 (23.3 27.4) | 1.3 (-12.5; 13.4) | 328 | 25.8 (23.5; 28.3) | | -0.7 (-16.1; 12.8) |
| 14–15M | 762 | 42.2 (39.9; 44.5) | 748 | 43.4 (41.1 45.8) | -2.9 (-14.0; 7.1) | 524 | 42.9 (40.1; 45.7) | | -1.7 (-13.8; 9.2) |
| 18–22M | 1103 | 63.0 (60.6; 65.2) | 1096 | 65.4 (63.1 67.7) | -3.9 (-13.1; 4.5) | 786 | 65.5 (62.7; 68.2) | | -4.0 (-14.1; 5.2) |
| ***Staphylococcus aureus*** | | | | | | | | | |
| 11–12M | 177 | 9.4 (8.2; 10.9) | 178 | 10.0 (8.6; 11.5) | -5.9 (-31.1; 14.5) | 133 | 10.5 (8.8; 12.3) | | -11.0 (-39.7; 12.1) |
| 14–15M | 390 | 21.6 (19.7; 23.6) | 393 | 22.8 (20.8; 24.9) | -5.7 (-21.9; 8.4) | 293 | 24.0 (21.6; 26.5) | | -11.1 (-29.6; 4.9) |
| 18–22M | 541 | 30.9 (28.7; 33.1) | 543 | 32.4 (30.2; 34.7) | -5.0 (-18.5; 7.0) | 395 | 32.9 (30.3; 35.7) | | -6.6 (-21.6; 6.6) |

N, maximum number of children with available results; n/%, number/percentage of children with new acquisition associated to the specified serotype/bacteria at the considered visit; M, months; TVC, total vaccinated cohort; CI, confidence interval.

**Supplementary Table 4. Cumulative acquisition of nasopharyngeal carriage and vaccine effectiveness from pre- to one month post-primary vaccination (Infant TVC for carriage - Immunogenicity subset)**

|  | **Infant control**  N=329 | | **Infant PHiD-CV 3+1**  N=243 | | | **Infant PHiD-CV 2+1**  N=245 | | |
| --- | --- | --- | --- | --- | --- | --- | --- | --- |
|  |  | **Cumulative acquisition rate** |  | **Cumulative acquisition rate** | **Vaccine effectiveness** |  | **Cumulative acquisition rate** | **Vaccine effectiveness** |
|  | n | % (95% CI) | n | % (95% CI) | % (95% CI) | n | % (95% CI) | % (95% CI) |
| ***Streptococcus pneumoniae*** | | | | | | | | |
| **Any vaccine serotype** | | | | | | | | |
| 6M | 27 | 8.2 (5.5; 11.7) | 16 | 6.6 (3.8; 10.5) | 19.8 (-54.4; 59.6) | 16 | 6.5 (3.8; 10.4) | 20.4 (-53.1; 59.9) |
| **Any serotype** | | | | | | | | |
| 6M | 53 | 16.1 (12.3; 20.5) | 42 | 17.3 (12.7; 22.6) | -7.3 (-63.9; 30.2) | 40 | 16.3 (11.9; 21.6) | -1.3 (-55.7; 34.5) |
| **Non-vaccine/non-vaccine-related serotypes** | | | | | | | | |
| 6M | 16 | 4.9 (2.8; 7.8) | 18 | 7.4 (4.4; 11.5) | -52.3 (-219.3; 26.7) | 18 | 7.3 (4.4; 11.4) | -51.1 (-216.7; 27.3) |
| **Vaccine-related serotype 6A** | | | | | | | | |
| 6M | 2 | 0.6 (0.1; 2.2) | 4 | 1.6 (0.5; 4.2) | -170.8 (-2893.5; 61.2) | 2 | 0.8 (0.1; 2.9) | -34.3 (-1752.6; 90.3) |
| **Vaccine-related serotype 19A** | | | | | | | | |
| 6M | 3 | 0.9 (0.2; 2.6) | 2 | 0.8 (0.1; 2.9) | 9.7 (-687.9; 92.5) | 1 | 0.4 (0.0; 2.3) | 55.2 (-457.5; 99.1) |
| **NTHi** | | | | | | | | |
| 6M | 7 | 2.1 (0.9; 4.3) | 8 | 3.3 (1.4; 6.4) | -54.7 (-401.2; 51.0) | 3 | 1.2 (0.3; 3.5) | 42.4 (-152.1; 90.4) |
| ***Moraxella catarrhalis*** | | | | | | | | |
| 6M | 48 | 14.6 (11.0; 18.9) | 40 | 16.5 (12.0; 21.7) | -12.8 (-75.3; 27.7) | 27 | 11.0 (7.4; 15.6) | 24.5 (-23.5; 54.7) |
| ***Staphylococcus aureus*** | | | | | | | | |
| 6M | 44 | 13.4 (9.9; 17.5) | 29 | 11.9 (8.1; 16.7) | 10.8 (-45.9; 46.2) | 30 | 12.2 (8.4; 17.0) | 8.4 (-49.0; 44.4) |

N, maximum number of children with available results; n/%, number/percentage of children with new acquisition associated to the specified serotype/bacteria at the considered visit; M, months; TVC, total vaccinated cohort; CI, confidence interval.

**Supplementary Table 5. Nasopharyngeal carriage at a given timepoint, and vaccine effectiveness per visit and overall; 7–11M catch-up groups (TVC for carriage)**

|  | **Control 7–11M**  **N=200** | | **PHiD-CV 7–11M**  **N=236** | | |
| --- | --- | --- | --- | --- | --- |
|  |  | **Colonization** |  | **Colonization** | **Vaccine effectiveness** |
|  | n | % (95% CI) | n | % (95% CI) | % (95% CI) |
| ***Streptococcus pneumoniae*** | | | | | |
| **Any vaccine serotype** | | | | | |
| 7–11M | 34 | 17.2 (12.2; 23.2) | 44 | 18.6 (13.9; 24.2) | - |
| 9–13M | 35 | 17.5 (12.5; 23.5) | 43 | 18.7 (13.9; 24.3) | -6.8 (-72.0; 33.2) |
| 13–17M | 55 | 27.9 (21.8; 34.7) | 43 | 19.1 (14.2; 24.9) | 31.5 (-3.9; 55.2) |
| 16–20M | 47 | 26.3 (20.0; 33.3) | 34 | 16.3 (11.5; 22.0) | 38.0 (1.6; 61.4) |
| 23–27M | 48 | 27.4 (21.0; 34.7) | 28 | 14.0 (9.5; 19.6) | 49.0 (17.0; 69.2) |
| *Across all visits* | *109* | *53.7 (46.6; 60.7)* | *96* | *40.0 (33.8; 46.5)* | *25.5 (1.1; 44.0)* |
| **Any serotype** | | | | | |
| 7–11M | 58 | 29.3 (23.1; 36.2) | 69 | 29.2 (23.5; 35.5) | - |
| 9–13M | 56 | 28.0 (21.9; 34.8) | 79 | 34.3 (28.2; 40.9) | -22.7 (-76.0; 14.0) |
| 13–17M | 87 | 44.2 (37.1; 51.4) | 81 | 36.0 (29.7; 42.6) | 18.5 (-11.6; 40.5) |
| 16–20M | 72 | 40.2 (33.0; 47.8) | 75 | 35.9 (29.4; 42.8) | 10.8 (-25.0; 36.3) |
| 23–27M | 75 | 42.9 (35.4; 50.5) | 68 | 34.0 (27.5; 41.0) | 20.7 (-11.6; 43.7) |
| *Across all visits* | *146* | *71.9 (65.2; 78.0)* | *154* | *64.2 (57.7; 70.2)* | *10.8 (-12.7; 29.3)* |
| **Non-vaccine/non-vaccine-related serotypes** | | | | | |
| 7–11M | 15 | 7.6 (4.3; 12.2) | 13 | 5.5 (3.0; 9.2) | - |
| 9–13M | 17 | 8.5 (5.0; 13.3) | 21 | 9.1 (5.7; 13.6) | -7.4 (-116.8; 46.0) |
| 13–17M | 23 | 11.7 (7.5; 17.0) | 25 | 11.1 (7.3; 16.0) | 4.8 (-75.5; 48.2) |
| 16–20M | 21 | 11.7 (7.4; 17.4) | 28 | 13.4 (9.1; 18.8) | -14.2 (-111.5; 37.4) |
| 23–27M | 21 | 12.0 (7.6; 17.8) | 26 | 13.0 (8.7; 18.5) | -8.3 (-102.5; 41.4) |
| *Across all visits* | *63* | *31.0 (24.7; 37.9)* | *71* | *29.6 (23.9; 35.8)* | *4.7 (-36.0; 33.1)* |
| **Vaccine-related serotype 6A** | | | | | |
| 7–11M | 4 | 2.0 (0.6; 5.1) | 5 | 2.1 (0.7; 4.9) | - |
| 9–13M | 4 | 2.0 (0.5; 5.0) | 7 | 3.0 (1.2; 6.2) | -52.2 (-608.9; 61.3) |
| 13–17M | 5 | 2.5 (0.8; 5.8) | 8 | 3.6 (10.5; 6.9) | -40.1 (-444.3; 59.6) |
| 16–20M | 1 | 0.6 (0.0; 3.1) | 6 | 2.9 (1.1; 6.1) | -413.9 (-23537.0; 37.7) |
| 23–27M | 2 | 1.1 (0.1; 4.1) | 6 | 3.0 (1.1; 6.4 | -162.5 (-2559.4; 53.1) |
| *Across all visits* | *12* | *5.9 (3.1; 10.1)* | *17* | *7.1 (4.2; 11.1)* | *-19.8 (-175.0; 46.1)* |
| **Vaccine-related serotype 19A** | | | | | |
| 7–11M | 3 | 1.5 (0.3; 4.4) | 3 | 1.3 (0.3; 3.7) | - |
| 9–13M | 0 | 0.0 (0.0; 1.8) | 3 | 1.3 (0.3; 3.8) | - |
| 13–17M | 0 | 0.0 (0.0; 1.9) | 1 | 0.4 (0.0; 2.5) | - |
| 16–20M | 2 | 1.1 (0.1; 4.0) | 3 | 1.4 (0.3; 4.1) | -28.5 (-1438.1; 85.3) |
| 23–27M | 1 | 0.6 (0.0; 3.1) | 4 | 2.0 (0.5; 5.0) | -250.0 (-17136.6; 65.4) |
| *Across all visits* | *4* | *2.0 (0.5; 5.0)* | *9* | *3.8 (1.7; 7.0)* | *-90.3 (-745.7; 46.9)* |
| **NTHi** | | | | | |
| 7–11M | 7 | 3.5 (1.4; 7.1) | 5 | 2.1 (0.7; 4.9) | - |
| 9–13M | 8 | 4.0 (1.7; 7.7) | 7 | 3.0 (1.2; 6.2) | 23.9 (-140.1; 76.5) |
| 13–17M | 12 | 6.1 (3.2; 10.4) | 19 | 8.4 (5.2; 12.9) | -38.6 (-213.2; 36.1) |
| 16–20M | 13 | 7.3 (3.9; 12.1) | 16 | 7.7 (4.4; 12.1) | -5.4 (-138.2; 52.5) |
| 23–27M | 13 | 7.4 (4.0; 12.4) | 20 | 10.0 (6.2; 15.0) | -34.6 (-194.5; 36.3) |
| *Across all visits* | *44* | *21.7 (16.2; 28.0)* | *62* | *25.8 (20.4; 31.9)* | *-19.2 (-79.6; 20.3)* |

N, maximum number of cultured swabs; n/%, number/percentage of children with swabs associated to the specified serotype/bacteria at the considered visit (or after at least 1 visit for “across all visits”); M, months (age); TVC, total vaccinated cohort; CI, confidence interval.

7–11M: pre-vaccination; 9–13M: 1 month post-primary vaccination; 13–17M: pre-booster; 16–20M, 3 months post-booster; 23–27M: 10 months post-booster.
Note: across-visits results include the pre-vaccination visit, and thus underestimate vaccine effectiveness.

**Supplementary Table 6: Nasopharyngeal carriage at a given timepoint, and vaccine effectiveness per visit and overall; 12–18M catch-up groups (TVC for carriage)**

|  | **Control 12–18M**  **N=1780** | | **PHiD-CV 12–18M**  **N=358** | | |
| --- | --- | --- | --- | --- | --- |
|  |  | **Colonization** |  | **Colonization** | **Vaccine effectiveness** |
|  | n | % (95% CI) | n | % (95% CI) | % (95% CI) |
| ***Streptococcus pneumoniae*** | | | | | |
| **Any vaccine serotype** | | | | | |
| 12–18M | 57 | 21.5 (16.7; 27.0) | 70 | 19.6 (15.6; 24.0) | - |
| 19–25M | 70 | 27.5 (22.1; 33.4) | 69 | 20.3 (16.1; 25.0) | 26.1 (-4.6; 47.8) |
| 21–27M | 53 | 20.9 (16.0; 26.4) | 64 | 18.9 (14.9; 23.5) | 9.3 (-33.2; 37.9) |
| *Across all visits* | *117* | *43.2 (37.2; 49.3)* | *129* | *35.3 (30.4; 40.5)* | *18.1 (-6.1;36.8)* |
| **Any serotype** | | | | | |
| 12–18M | 88 | 33.2 (27.6; 39.2) | 125 | 34.9 (30.0; 40.1) | - |
| 19–25M | 112 | 43.9 (37.7; 50.2) | 152 | 44.7 (39.3; 50.2) | -1.8 (-31.1; 20.8) |
| 21–27M | 105 | 41.3 (35.2; 47.7) | 132 | 39.1 (33.8; 44.5) | 5.5 (-23.3; 27.4) |
| *Across all visits* | *176* | *64.9 (58.9; 70.6)* | *236* | *64.7 (59.5; 69.6)* | *0.4 (-21.7; 18.4)* |
| **Non-vaccine/non-vaccine-related serotypes** | | | | | |
| 12–18M | 21 | 7.9 (5.0; 11.9) | 32 | 8.9 (6.2; 12.4) | - |
| 19–25M | 27 | 10.6 (7.1; 15.0) | 50 | 14.7 (11.1; 18.9) | -38.9 (-130.7; 14.7) |
| 21–27M | 30 | 11.8 (8.1; 16.4) | 48 | 14.2 (10.7; 18.4) | -20.2 (-96.5; 25.4) |
| *Across all visits* | *61* | *22.5 (17.7; 28.0)* | *91* | *24.9 (20.6; 29.7)* | *-10.8 (-55.8; 20.8)* |
| **Vaccine-related serotype 6A** | | | | | |
| 12–18M | 6 | 2.3 (0.8; 4.9) | 14 | 3.9 (2.2; 6.5) | - |
| 19–25M | 10 | 3.9 (1.9; 7.1) | 26 | 7.6 (5.1; 11.0) | -95.0 (-353.2; 9.0) |
| 21–27M | 12 | 4.7 (2.5; 8.1) | 15 | 4.4 (2.5; 7.2) | 6.1 (-119.8; 59.0) |
| *Across all visits* | *20* | *7.4 (4.6; 11.2)* | *42* | *11.5 (8.4; 15.2)* | *-55.9 (-180.3; 10.5)* |
| **Vaccine-related serotype 19A** | | | | | |
| 12–18M | 0 | 0.0 (0.0; 1.4) | 2 | 0.6 (0.1; 2.0) | - |
| 19–25M | 3 | 1.2 (0.2; 3.4) | 2 | 0.6 (0.1; 2.1) | 50.0 (-336.5; 95.8) |
| 21–27M | 3 | 1.2 (0.2; 3.4) | 2 | 0.6 (0.1; 2.1) | 49.9 (-337.3; 95.8) |
| *Across all visits* | *6* | *2.2 (0.8; 4.8)* | *5* | *1.4 (0.4; 3.2)* | *38.1 (-143.3; 85.1)* |
| **NTHi** | | | | | |
| 12–18M | 12 | 4.5 (2.4; 7.8) | 20 | 5.6 (3.4; 8.5) | - |
| 19–25M | 18 | 7.1 (4.2; 10.9) | 20 | 5.9 (3.6; 8.9) | 16.7 (-67.1; 58.1) |
| 21–27M | 25 | 9.8 (6.5; 14.2) | 26 | 7.7 (5.1; 11.1) | 21.8 (-41.1; 56.6) |
| *Across all visits* | *49* | *18.1 (13.7; 23.2)* | *56* | *15.3 (11.8; 19.5)* | *15.1 (-27.1; 43.2)* |

N, maximum number of cultured swabs; n/%, number/percentage of children with swabs associated to the specified serotype/bacteria at the considered visit (or after at least 1visit for “across all visits”); M, months (age); TVC, total vaccinated cohort; CI, confidence interval.

12–18M: pre-vaccination; 19–25M: 1 month post-dose 2; 21–27M: 3 months post-dose 2.

Note: across-visits results include the pre-vaccination visit, and thus underestimate vaccine effectiveness.

**Supplementary Table 7. Percentages of children with serotype-specific antibody concentration ≥0.2 µg/mL (22F-ELISA), anti-Protein D antibody concentration >100 EL.U/mL, and antibody GMCs (ATP cohort for immunogenicity)**

|  |  | **Infant PHiD-CV 3+1**  N=209 | | | | **Infant PHiD-CV 2+1**  N=205 | | | | | | | **Catch-up PHiD-CV 7–11M**  N=151 | | | | | | **Catch-up PHiD-CV 12–18M**  N=167 | | | |
| --- | --- | --- | --- | --- | --- | --- | --- | --- | --- | --- | --- | --- | --- | --- | --- | --- | --- | --- | --- | --- | --- | --- |
|  |  | n | % ≥0.2 µg/mL  (95% CI) | | GMC  (95% CI) | n | % ≥0.2 µg/mL  (95% CI) | | | | GMC  (95% CI) | | n | | % ≥0.2 µg/mL  (95% CI) | | | GMC  (95% CI) | n | % ≥0.2 µg/mL  (95% CI) | | GMC  (95% CI) |
| *Vaccine serotypes* | | | | | | | | | | | | | | | | | | | | | | |
| 1 | Post-primary | 208 | 100  (98.2; 100) | | 1.86  (1.68; 2.05) | 201 | | 98.0  (95.1; 99.5) | | | 1.37  (1.25; 1.52) | 149 | | | | 98.7  (95.3; 99.8) | | 1.96  (1.72; 2.23) | 166 | | 99.4  (96.7; 100) | 1.87  (1.67; 2.09) |
|  | Post-booster | 189 | 100  (98.1; 100) | | 2.13  (1.88; 2.41) | 193 | | 100  (98.1; 100) | | | 1.91  (1.72; 2.12) | 137 | | | | 100  (97.3; 100) | | 2.62  (2.33; 2.94) |  | | - | - |
| 4 | Post-primary | 207 | 99.5  (97.4; 100) | | 2.47  (2.23; 2.75) | 202 | | 99.0  (96.5; 99.9) | | | 1.87  (1.68; 2.07) | 150 | | | | 100  (97.6; 100) | | 5.85  (5.16; 6.63) | 167 | | 100  (97.8; 100) | 5.28  (4.77; 5.84) |
|  | Post-booster | 189 | 100  (98.1; 100) | | 3.61  (3.20; 4.06) | 193 | | 100  (98.1; 100) | | | 3.16  (2.84; 3.52) | 135 | | | | 100  (97.3; 100) | | 5.45  (4.85; 6.14) |  | | - | - |
| 5 | Post-primary | 208 | 100  (98.2; 100) | | 2.73  (2.47; 3.01) | 201 | | 98.5  (95.8; 99.7) | | | 1.97  (1.76; 2.19) | 151 | | | | 100  (97.6; 100) | | 2.40  (2.13; 2.72) | 167 | | 100  (97.8; 100) | 3.45  (3.05; 3.90) |
|  | Post-booster | 188 | 99.5  (97.1; 100) | | 3.27  (2.87; 3.73) | 193 | | 100  (98.1; 100) | | | 2.82  (2.52; 3.15) | 137 | | | | 100  (97.3; 100) | | 4.11  (3.71; 4.56) |  | | - | - |
| 6B | Post-primary | 165 | 79.3  (73.2; 84.6) | | 0.51  (0.43;0.62) | 136 | | 66.3  (59.4; 72.8) | | | 0.32  (0.27; 0.37) | 91 | | | | 60.3  (52.0; 68.1) | | 0.27  (0.21; 0.33) | 144 | | 86.2  (80.1; 91.1) | 0.69  (0.57; 0.83) |
|  | Post-booster | 179 | 94.7  (90.5; 97.4) | | 1.43  (1.22; 1.68) | 187 | | 96.9  (93.4; 98.9) | | | 1.43  (1.25; 1.65) | 124 | | | | 90.5  (84.3; 94.9) | | 1.06  (0.85; 1.31) |  | | - | - |
| 7F | Post-primary | 209 | 100  (98.3; 100) | | 2.90  (2.62; 3.20) | 202 | | 98.5  (95.8; 99.7) | | | 1.76  (1.57; 1.97) | 150 | | | | 100  (97.6; 100) | | 3.61  (3.21; 4.06) | 167 | | 100  (97.8; 100) | 3.95  (3.58; 4.35) |
|  | Post-booster | 189 | 100  (98.1; 100) | | 4.25  (3.80; 4.75) | 193 | | 100  (98.1; 100) | | | 3.62  (3.28; 4.01) | 137 | | | | 100  (97.3; 100) | | 5.44  (4.80; 6.15) |  | | - | - |
| 9V | Post-primary | 207 | 99.5  (97.4; 100) | | 2.23  (2.00; 2.48) | 201 | | 98.0  (95.1; 99.5) | | | 1.38  (1.24; 1.54) | 146 | | | | 96.7  (92.4; 98.9) | | 1.42  (1.24; 1.64) | 166 | | 99.4  (96.7; 100) | 1.60  (1.42; 1.81) |
|  | Post-booster | 188 | 100  (98.1; 100) | | 3.98  (3.56; 4.46) | 193 | | 100  (98.1; 100) | | | 3.88  (3.47; 4.33) | 137 | | | | 100  (97.3; 100) | | 2.81  (2.44; 3.23) |  | | - | - |
| 14 | Post-primary | 209 | 100  (98.3; 100) | | 5.00  (4.46; 5.61) | 202 | | 98.5  (95.8; 99.7) | | | 3.31  (2.92; 3.75) | 150 | | | | 100  (97.6; 100) | | 3.81  (3.34; 4.35) | 167 | | 100  (97.8; 100) | 6.04  (5.37; 6.79) |
|  | Post-booster | 189 | 100  (98.1; 100) | | 6.40  (5.62; 7.29) | 192 | | 99.5  (97.1; 100) | | | 4.84  (4.26; 5.51) | 137 | | | | 100  (97.3; 100) | | 8.38  (7.42; 9.47) |  | | - | - |
| 18C | Post-primary | 207 | 99.0  (96.6; 99.9) | | 6.51  (5.63; 7.54) | 203 | | 99.0  (96.5; 99.9) | | | 3.38  (2.88; 3.95) | 149 | | | | 99.3  (96.3; 100) | | 10.03  (8.67; 11.61) | 166 | | 100  (97.8; 100) | 21.27  (18.70; 24.19) |
|  | Post-booster | 187 | 98.9  (96.2; 99.9) | | 10.43  (8.94; 12.18) | 193 | | 100  (98.1; 100) | | | 10.60  (9.48; 11.84) | 137 | | | | 100  (97.3; 100) | | 19.87  (17.08; 23.12) |  | | - | - |
| 19F | Post-primary | 206 | 98.6  (95.9; 99.7) | | 5.91  (5.06; 6.89) | 200 | | 97.6  (94.4; 99.2) | | | 3.40  (2.92; 3.97) | 147 | | | | 97.4  (93.4; 99.3) | | 6.64  (5.41; 8.15) | 166 | | 100  (97.8; 100) | 12.10  (10.38; 14.11) |
|  | Post-booster | 189 | 100  (98.1; 100) | | 8.04  (7.04; 9.17) | 192 | | 99.5  (97.1; 100) | | | 7.41  (6.54; 8.40) | 136 | | | | 99.3  (96.0; 100) | | 11.73  (9.73; 14.13) |  | | - | - |
| 23F | Post-primary | 175 | 84.1  (78.4; 88.8) | | 0.68  (0.56; 0.83) | 158 | | 77.1  (70.7; 82.6) | | | 0.54  (0.45; 0.65) | 117 | | | | 77.5  (70.0; 83.9) | | 0.55  (0.44; 0.70) | 157 | | 94.0  (89.3; 97.1) | 1.27  (1.07; 1.50) |
|  | Post-booster | 179 | 94.7  (90.5; 97.4) | | 2.30  (1.90; 2.77) | 188 | | 97.4  (94.1; 99.2) | | | 2.18  (1.88; 2.54) | 134 | | | | 97.8  (93.7; 99.5) | | 2.04  (1.71; 2.43) |  | | - | - |
| *Vaccine-related serotypes* | | | | | | | | | | | | | | | | | | | | | | |
| 6A | Post-primary | 80 | 38.5  (31.8; 45.4) | | 0.13  (0.11; 0.15) | 57 | | | 28.1  (22.0; 34.8) | | 0.09  (0.08; 0.11) | | 56 | | | | 37.3  (29.6; 45.6) | 0.11  (0.09; 0.14) | 106 | | 63.5  (55.7; 70.8) | 0.32  (0.26; 0.41) |
|  | Post-booster | 145 | 76.7  (70.0; 82.5) | | 0.53  (0.43; 0.65) | 150 | | | 77.7  (71.2; 83.4) | | 0.50  (0.42; 0.60) | | 113 | | | | 82.5  (75.1; 88.4) | 0.70  (0.55; 0.90) |  | | - | - |
| 19A | Post-primary | 86 | 41.3  (34.6; 48.4) | | 0.15  (0.12; 0.18) | 86 | | | 42.2  (35.3; 49.3) | | 0.13  (0.11; 0.16) | | 94 | | | | 62.3  (54.0; 70.0) | 0.33  (0.26; 0.42) | 160 | | 95.8  (91.6; 98.3) | 2.61  (2.12; 3.22) |
|  | Post-booster | 158 | 84.0  (78.0; 89.0) | | 0.95  (0.75; 1.19) | 167 | | | 86.5  (80.9; 91.0) | | 0.89  (0.74; 1.07) | | 128 | | | | 93.4  (87.9; 97.0) | 1.98  (1.53; 2.56) |  | | - | - |
| *Protein D* | | | | | | | | | | | | | | | | | | | | | | |
|  |  | n | % ≥100 EL.U/mL  (95% CI) | GMC  (95% CI) | | n | | | | % ≥100 EL.U/mL  (95% CI) | GMC  (95% CI) | | |  |  |  |  |  |  |  |  |  |
|  | Post-primary | 208 | 99.5  (97.4; 100) | 1869.4  (1670.7; 2091.7) | | 202 | | | | 99.5  (97.3; 100) | 1062.9  (936.0; 1207.0) | | |  |  |  |  |  |  |  |  |  |
|  | Post-booster | 188 | 100  (98.1; 100) | 2734.7  (2406.0; 3108.3) | | 192 | | | | 99.5  (97.1; 100) | 1903.9  (1642.7; 2206.6) | | |  |  |  |  |  |  |  |  |  |

N, maximum number of children with available results; n/%, number/percentage of children with antibody concentrations ≥0.2 µg/mL (or ≥100 EL.U/mL for protein D); GMC, geometric mean concentration; EL.U, ELISA units; CI, confidence interval; M, months; ATP, according-to-protocol.
Infant 3+1, 3 primary doses and booster; infant 2+1, 2 primary doses and booster; catch-up PHiD-CV 7–11M; 2 primary doses and booster; catch-up PHiD-CV 12–18M, 2 doses.

**Supplementary Table 8. Opsonophagocytic activity results for the infant cohort (Infant ATP cohort for immunogenicity)**

|  | |  | **PHiD-CV 3+1**  N=202 | | | **PHID-CV 2+1**  N=196 | | | |
| --- | --- | --- | --- | --- | --- | --- | --- | --- | --- |
|  | |  | n | % ≥8  (95% CI) | GMT  (95% CI) | n | % ≥8  (95% CI) | GMT  (95% CI) | |
| *Vaccine serotypes* | | | | | | | |  |  |
| 1 | | Post-primary | 145 | 71.8 (65.0; 77.9) | 52.8 (40.7; 68.4) | 131 | 66.8 (59.8; 73.4) | 38.3 (30.0; 49.0) | |
|  | | Post-booster | 173 | 94.0 (89.6; 97.0) | 305.6 (238.9; 390.8) | 160 | 86.5 (80.7; 91.1) | 256.9 (194.6; 339.2) | |
| 4 | | Post-primary | 199 | 100 (98.2; 100) | 845.6 (746.8; 957.4) | 191 | 99.5 (97.1; 100) | 553.0 (484.9; 630.5) | |
|  | | Post-booster | 184 | 100 (98.0; 100) | 1745.7 (1476.3; 2064.1) | 186 | 99.5 (97.1; 100) | 1143.4 (961.9; 1359.0) | |
| 5 | | Post-primary | 185 | 93.0 (88.5; 96.1) | 65.9 (55.8; 77.7) | 172 | 88.2 (82.8; 92.4) | 48.5 (40.5; 58.0) | |
|  | | Post-booster | 179 | 96.8 (93.1; 98.8) | 191.6 (155.9; 235.4) | 179 | 96.2 (92.4; 98.5) | 145.6 (120.2; 176.2) | |
| 6B | | Post-primary | 175 | 89.7 (84.6; 93.6) | 740.6 (558.3; 982.4) | 149 | 80.1 (73.6; 85.6) | 268.6 (193.3; 373.3) | |
|  | | Post-booster | 171 | 94.5 (90.1; 97.3) | 736.3 (576.2; 941.0) | 176 | 96.2 (92.3; 98.4) | 879.1 (695.4; 1111.2) | |
| 7F | | Post-primary | 197 | 100 (98.1; 100) | 3894.8 (3320.2; 4569.0) | 189 | 99.5 (97.1; 100) | 2553.5 (2124.7; 3069.0) | |
|  | | Post-booster | 184 | 100 (98.0; 100) | 5219.7 (4440.2; 6136.0) | 185 | 100 (98.0; 100) | 4863.2 (4211.1; 5616.3) | |
| 9V | | Post-primary | 194 | 100 (98.1; 100) | 2798.0 (2411.9; 3246.0) | 186 | 100 (98.0; 100) | 1687.2 (1442.7; 1973.1) | |
|  | | Post-booster | 183 | 100 (98.0; 100) | 3491.2 (3049.2; 3997.3) | 179 | 100 (98.0; 100) | 3196.0 (2718.4; 3757.6) | |
| 14 | | Post-primary | 197 | 99.5 (97.2; 100) | 1831.3 (1572.5; 2132.7) | 188 | 98.4 (95.5; 99.7) | 1146.3 (944.2; 1391.8) | |
|  | | Post-booster | 185 | 100 (98.0; 100) | 2657.2 (2280.6; 3096.1) | 187 | 100 (98.0; 100) | 1724.2 (1475.5; 2014.8) | |
| 18C | | Post-primary | 188 | 97.9 (94.8; 99.4) | 543.3 (444.5; 664.2) | 168 | 91.3 (86.3; 94.9) | 230.6 (177.0; 300.4) | |
|  | | Post-booster | 181 | 98.9 (96.1; 99.9) | 1066.1 (890.3; 1276.6) | 183 | 100 (98.0; 100) | 1052.2 (881.8; 1255.5) | |
| 19F | | Post-primary | 191 | 97.4 (94.1; 99.2) | 649.6 (522.7; 807.4) | 163 | 87.2 (81.5; 91.6) | 197.6 (148.6; 262.8) | |
|  | | Post-booster | 177 | 96.7 (93.0; 98.8) | 1026.0 (807.3; 1303.9) | 180 | 96.8 (93.1; 98.8) | 854.6 (672.1; 1086.6) | |
| 23F | | Post-primary | 182 | 92.9 (88.3; 96.0) | 1900.7 (1440.0; 2508.7) | 168 | 89.4 (84.0; 93.4) | 897.1 (663.5; 1212.9) | |
|  | | Post-booster | 183 | 99.5 (97.0; 100) | 3248.2 (2705.9; 3899.2) | 179 | 97.3 (93.8; 99.1) | 2630.7 (2047.9; 3379.2) | |
| *Vaccine-related serotypes* | | | | | | | |  |  |
| 6A | | Post-primary | 137 | 71.7 (64.8; 78.0) | 90.8 (66.4; 124.3) | 104 | 56.8 (49.3; 64.1) | 43.1 (31.2; 59.5) | |
|  | | Post-booster | 138 | 78.0 (71.1; 83.8) | 173.8 (125.7; 240.4) | 141 | 83.9 (77.5; 89.1) | 285.9 (205.3; 398.2) | |
| 19A | | Post-primary | 82 | 42.5 (35.4; 49.8) | 25.2 (18.3; 34.8) | 55 | 28.9 (22.6; 36.0) | 11.9 (9.2; 15.5) | |
|  | | Post-booster | 142 | 78.5 (71.7; 84.2) | 145.0 (104.7; 200.9) | 127 | 69.4 (62.2; 76.0) | 78.9 (55.5; 112.2) | |

N, maximum number of children with available results; n/%, number/percentage of children with antibody titers ≥8; OPA, opsonophagocytic assay; GMT, geometric mean titer; CI, confidence interval; ATP, according-to-protocol.
Infant 3+1, 3 primary doses and booster; infant 2+1, 2 primary doses and booster. Opsonophagocytic assays were not performed for the catch-up cohorts.

**Supplementary Table 9. Unsolicited adverse events and serious adverse events (TVC for safety)**

| 1. *Percentage of doses for which* ≥*1 unsolicited AE was reported* | | | | | |
| --- | --- | --- | --- | --- | --- |
| ***Infant cohort:*** |  | **PHiD-CV 3+1**  5501 doses | **Control 3+1**  3186 doses | **PHiD-CV 2+1**  2610 doses | **Control 2+1**  1709 doses |
| **Unsolicited AEs** | Post-primary | 32.9% | 26.3% | 30.0% | 24.0% |
|  | Post-booster | 29.2% | 26.6% | 28.5% | 29.2% |
|  |  |  |  |  |  |
| **Vaccination-related* unsolicited AEs** | Post-primary | 16.9% | 8.3% | 16.4% | 7.1% |
|  | Post-booster | 12.4% | 7.9% | 15.2% | 7.9% |
|  |  |  |  |  |  |
| **Grade 3 unsolicited AEs** | Post-primary | 2.2% | 2.5% | 2.2% | 3.0% |
|  | Post-booster | 3.7% | 3.7% | 3.0% | 4.8% |
|  |  |  |  |  |  |
|  |  |  |  |  |  |
| ***Catch-up cohort:*** |  | **7–11M PHiD-CV**  474 doses | **7–11M Control**  404 doses | **12–18M PHiD-CV**  717 doses | **12–18M Control**  537 doses |
| **Unsolicited AEs** | Post-primary | 46.4% | 47.0% | 38.5% | 42.3% |
|  | Post-booster | 22.6% | 24.4% | - | - |
|  |  |  |  |  |  |
| **Vaccination-related unsolicited AEs*** | Post-primary | 19.4% | 10.6% | 17.2% | 11.5% |
|  | Post-booster | 9.3% | 5.1% | - | - |
|  |  |  |  |  |  |
| **Grade 3 unsolicited AEs** | Post-primary | 7.4% | 9.4% | 4.3% | 5.8% |
|  | Post-booster | 4.0% | 4.6% | - | - |
|  |  |  |  |  |  |
| 1. *Number of children reporting at least 1 serious adverse event* | | | | | |
| ***Infant cohort:*** |  | **PHiD-CV 3+1**  N=1849 | **Control 3+1**  N=1069 | **PHiD-CV 2+1**  N=1316 | **Control 2+1**  N=859 |
| **SAEs:** n (%) |  | 163 (8.8%) | 77 (7.2%) | 96 (7.3%) | 74 (8.6%) |
| **Vaccination-related* SAEs:** n | | 4 | 1 | 0 | 1 |
|  |  |  |  |  |  |
| ***Catch-up cohort:*** |  | **7–11M PHiD-CV**  N=241 | **7–11M Control**  N=204 | **12–18M PHiD-CV**  N=368 | **12–18M Control**  N=271 |
| **SAEs:** n (%) |  | 24 (10%) | 18 (8.8%) | 23 (6.3%) | 14 (5.2%) |
| **Vaccination-related* SAEs:** n | | 0 | 0 | 0 | 0 |

N, number of children per group; %, percentage of doses for which at least 1 unsolicited AE was reported in the 31-day post-vaccination period; n (%), number (percentage) of children reporting at least 1 SAE; AE, adverse event, SAE; serious adverse event. *Considered by the investigator to be related to vaccination.

**Supplementary Methods**

*Assessment of nasopharyngeal carriage*

Nasopharyngeal samples were cultured on sheep blood, sheep blood with gentamicin (5 µg/ml), and chocolate agar plates. The plates were incubated in 5% CO_2_ at 35–37°C for 18 to 24 hours and colonies suspected of being *Streptococcus pneumoniae, Haemophilus influenzae, Moraxella catarrhalis, Staphylococcus aureus* and *Streptococcus pyogenes* were identified using generally accepted methods as described earlier [[1](#_ENREF_1)]*.* All isolated bacterial strains were stored in 10% skim milk medium (Difco™, Skim milk) at below -65°C. Identified *S. pneumoniae* isolates were serotyped, as a mixture containing from one up to four optochin sensitive strains, with pneumococcal antisera (Statens Serum Institut, Denmark) for the neutral serogroups 7 and 14 by latex agglutination, followed by counter-immunoelectrophoresis and as a confirmation method by Quellung reaction, when needed. All serotypes within serogroups were differentiated using pneumococcal factor antisera; e.g. within *S. pneumoniae* serogroup 6, serotype 6C was differentiated from serotype 6A, 6B and 6D. *H. influenzae* isolates were serotyped with polyvalent and type-specific antisera (Difco™) by counter-immunoelectrophoresis, and underwent further discrimination from *Haemophilus haemolyticus* species using a GlaxoSmithKline *in-house* duplex PCR assay at GlaxoSmithKline Vaccines laboratories.

*Immunogenicity assessment*

Pneumococcal serotype-specific IgG antibodies against PHiD-CV serotypes 1, 4, 5, 6B, 7F, 9V, 14, 18C, 19F and 23F, and against vaccine-related serotypes 6A and 19A were measured using GlaxoSmithKline Vaccines’ enzyme-linked immunosorbent assay (22F-ELISA; assay cut-off, 0.05 µg/mL). Immune responses were presented in terms of the percentages of children with antibody concentrations ≥0.2 µg/mL (this level is equivalent to the antibody concentration of 0.35 µg/mL measured by the non-22F ELISA of the WHO reference laboratory) [[2](#_ENREF_2)].

Opsonophagocytic activity (OPA) against the above-mentioned serotypes was measured by a pneumococcal killing assay using an HL-60 cell line, with an opsonic titer cut-off value of 8. The results are presented as the reciprocal of the dilution of serum (opsonic titer) able to sustain 50% killing of pneumococci under the assay conditions.

Anti-protein D antibodies were quantified using an ELISA assay developed by GlaxoSmithKline Vaccines, with a cut-off of 100 ELISA units per mL.

*Safety assessment*

Solicited local and general symptoms were recorded within 4 days post-vaccination, unsolicited adverse events (AEs) within 31 days post-vaccination. Fever was defined as a rectal temperature ≥38°C or oral/axillary/tympanic temperature ≥37.5°C. Symptom intensity was graded on a scale of 1 (mild) to 3 (severe). Grade 3 for redness and swelling at the injection site meant redness or swelling with a diameter >30 mm; for pain, crying when the limb was moved or the limb being spontaneously painful; for irritability, crying that could not be comforted or prevented normal activity; for loss of appetite, not eating at all; for fever, rectal temperature >40°C or oral/axillary/tympanic temperature >39.5°C; and for all other AEs, preventing normal everyday activities.

Serious adverse events, defined as any medical occurrence that resulted in death, disability or incapacity, was life-threatening or required hospitalization, were recorded over the whole study period.

**References**

1. Kilpi T, Herva E, Kaijalainen T, Syrjanen R, Takala AK. Bacteriology of acute otitis media in a cohort of Finnish children followed for the first two years of life. Pediatr Infect Dis J **2001**;20(7):654-62.

2. Poolman JT, Frasch CE, Kayhty H, Lestrate P, Madhi SA, Henckaerts I. Evaluation of pneumococcal polysaccharide immunoassays using a 22F adsorption step with serum samples from infants vaccinated with conjugate vaccines. Clin Vaccine Immunol **2010**;17(1):134-42.
